# MiR-191 Regulates Primary Human Fibroblast Proliferation and Directly Targets Multiple Oncogenes

**DOI:** 10.1371/journal.pone.0126535

**Published:** 2015-05-20

**Authors:** Damon Polioudakis, Nathan S. Abell, Vishwanath R. Iyer

**Affiliations:** Center for Systems and Synthetic Biology, Institute for Cellular and Molecular Biology, Department of Molecular Biosciences, University of Texas at Austin, Austin, Texas, United States of America; University of Barcelona, SPAIN

## Abstract

miRNAs play a central role in numerous pathologies including multiple cancer types. miR-191 has predominantly been studied as an oncogene, but the role of miR-191 in the proliferation of primary cells is not well characterized, and the miR-191 targetome has not been experimentally profiled. Here we utilized RNA induced silencing complex immunoprecipitations as well as gene expression profiling to construct a genome wide miR-191 target profile. We show that miR-191 represses proliferation in primary human fibroblasts, identify multiple proto-oncogenes as novel miR-191 targets, including CDK9, NOTCH2, and RPS6KA3, and present evidence that miR-191 extensively mediates target expression through coding sequence (CDS) pairing. Our results provide a comprehensive genome wide miR-191 target profile, and demonstrate miR-191’s regulation of primary human fibroblast proliferation.

## Introduction

The ability of mammalian cells to transition from a quiescent to a proliferative state is a fundamental aspect of normal cell biology, and forms the basis for diverse physiological processes such as lymphocyte activation, hepatocyte regeneration, and wound healing [1–4]. However, the capacity to chronically sustain proliferative signaling is essential for tumorigenesis and is a hallmark of cancer [5]. Cancer cells display numerous other physiological abnormalities, typically resistance to apoptosis, angiogenesis, and invasion and metastasis; but cancer is often considered to be foremost a disease of the cell cycle [5]. The pathways that control proliferation in normal cells are generally perturbed in cancer, and many cell cycle regulators that control reentry and progression through the cell cycle are altered in cancer cells [5, 6].

Primary human dermal fibroblasts are an exceptional model to study the genetic pathways that regulate proliferation in natural physiological processes and also in cancer progression. Primary fibroblasts may be induced to enter or exit a quiescent state in response to exposure to or deprivation of serum containing growth factors respectively. Fibroblast proliferation plays a key role in wound healing, and serum stimulation of fibroblasts produces a genetic program similar to that activated during the wound healing process [7]. Physiologically, the wound response is similar to cancer progression; wounding activates signaling cascades that lead to epithelial and fibroblast cell proliferation, matrix remodeling, cell migration, and angiogenesis [7]. Cancer and the wound response also share genetic similarities, as many of the genes differentially expressed in fibroblasts following serum stimulation are also differentially expressed in tumor cells, associated fibroblasts or both, and include a number of key cell cycle regulators [8]. In addition, the expression profiles of proliferating fibroblasts are good predictors of cancer progression [8]. Multiple protein coding genes and miRNAs are differentially expressed between proliferating and quiescent fibroblasts [9].

miRNAs are short noncoding RNAs that regulate gene expression post transcriptionally by a combination of inhibition of translation initiation and mRNA destabilization. More than 60% of human protein coding genes are under selective pressure to maintain 3’ UTR pairing to miRNAs, indicative of their widespread control of biological processes including differentiation, proliferation, migration, and apoptosis [10, 11]. In addition to their widespread role in normal physiological processes, miRNAs are involved in numerous pathologies and play central roles in tumorigenesis [12–14]. Multiple miRNAs are known to function as oncogenes and/or tumor suppressors, and miRNAs are located at 50% of all fragile regions or sites showing copy number alterations in cancer [15].

miR-191 has been shown to play a role in multiple cancer types, including gastric, colorectal, breast, thyroid, and hepatocellular carcinoma [16–20]. Proliferation related targets have been identified for miR-191, such as CDK6 and SATB1 [21]. Despite the clear link between miR-191, proliferation, and tumorigenesis, the regulation of proliferation by miR-191 has not been explored in primary cells, and genome wide target identification for miR-191 has not been performed with current biochemical techniques.

In this study, we investigated the regulation of cell proliferation in primary human fibroblasts by miR-191. We experimentally identified the targets of miR-191 by conducting extensive profiling of RNA induced silencing complex (RISC) associated transcripts in combination with gene expression profiling. GO-term enrichment analysis of these targets identified multiple genes involved in proliferation and cell cycle regulation, and we experimentally confirmed multiple proto-oncogenes as direct targets of miR-191.

## Materials and Methods

### Normal Cell Culture Conditions

Primary human foreskin fibroblasts (ATCC CRL #2091) and HeLa cells were cultured in Dulbecco’s Modified Eagle’s Medium (DMEM) Supplemented with 10% fetal bovine serum (FBS) at 37°C 5% CO_2_.

### RNA Oligos and Transfections

miRNA guide and anti-guide mature sequences, guide and anti-guide sequences for an siRNA against GFP (Control siRNA), and sequences for a scrambled control siRNA (Control siRNA 2) were obtained from miRBase (www.mirbase.org/), [22], and [23] respectively. miR-191 guide: 5’-CAACGGAAUCCCAAAAGCAGCUG-3’ and anti-guide: 5’GCUGCGCUUGGAUUUCGUCCCC-3’; Control RNA 1 guide: 5’-CUGGAGUUGUCCCAAUUCCUU-3’ and anti-guide: 5’-AGAAUUGGGACAACUCCAGUU-3’; and Control RNA 2 guide: 5’-AAUUCUCCGAACGUGUCACGUUA-3’ and anti-guide: 5’-ACGUGACACGUUCGGAGAAUUCA-3’. Guide and corresponding anti-guide RNA oligos from IDT were annealed in RNA annealing buffer (20 mM HEPES, pH 7.3, 50 mM KCl, 2 mM MgCl_2_) to form mature miRNA or siRNA duplexes. The miRNA and Control siRNA oligos have the same chemical modifications, a 5’ phosphate and 3’ OH. Unless otherwise noted, the RNA duplexes were transfected at a final concentration of 100 nM using Lipofectamine 2000 according to the manufacturer’s instructions. The miRNA inhibitors and control were miRCURY Locked Nucleic Acid (LNA) miRNA Inhibitors and Negative Control Inhibitor obtained from Exiqon and transfected at a final concentration of 10 nM using Lipofectamine 2000.

### Cell Counting Assays

For the transient miRNA overexpression experiment, fibroblasts were seeded at 10,000 cells per well in 6-well plates. Cells were grown 24 hours, and then transiently transfected with miR-191 or Control siRNA duplexes. 0, 24, 48, 72, and 96 hours post transfection, cells were trypsinized, and counted in a hemacytometer with 9 fields averaged for each biological replicate. For the miRNA inhibition experiment, fibroblasts were seeded at 10,000 cells per well in 6-well plates. Cells were grown 24 hours, then washed 3X with PBS and media was replaced with DMEM 0.1% FBS. In conjunction with media replacement, cells were transiently transfected with miR-191 miRCURY Locked Nucleic Acid (LNA) miRNA Inhibitor or Negative Control Inhibitor obtained from Exiqon (10 nM final concentration). 0, 24, and 48 hours after transfection, cells were trypsinized, counted in a hemacytometer, and 9 fields were averaged for each biological replicate.

### Flow Cytometry

Fibroblasts were seeded at 50,000 cells per well in 6-well plates, and cultured in DMEM Supplemented with 10% FBS. Cells were grown 24 hours, and then transiently transfected with miR-191, Control siRNA, or Control siRNA 2 duplexes (100 nM final concentration). For M-phase trapping experiments, 125 ng/ml nocodazole was added at the time of transfection. 24 hours post transfection, cells were trypsinized, washed with PBS, and fixed for 24 hours in 70% ethanol at -20°C. For Ki67 staining, following ethanol fixation, cells were washed with Stain Buffer (BD Pharmingen), incubated 30 minutes with FITC Mouse Anti-Human Ki67 antibody (BD Pharmingen), washed, and resuspended in 500 μl Stain Buffer with Propidium Iodide (PI) Staining Solution (5 μg/mL) (BD Pharmingen). For PI staining, following ethanol fixation, cells were washed twice with PBS, and then resuspended in PI staining solution (50ug/mL PI with 100ug/mL RNase in PBS). Flow cytometry analysis for Ki67 in primary human fibroblasts was done using a FACsCalibur flow cytometer and 10,000 events above threshold levels were counted for each sample (BD Biosciences). Data analysis was done using FlowJo.

### RISC Immunoprecipitation

To immunoprecipitate RISC-RNA complexes we adapted the protocol from Hendrickson *et al*. [24]. HeLa cells were cultured in 10 cm^2^ tissue culture plates and transiently transfected with either miRNA mature duplexes or Control RNA duplexes at a final concentration of 100 nM or mock transfected. 24 hours post transfection, cells were washed 2X with PBS, 0.5 mL of lysis buffer was added to the plate, followed by incubation at 4°C for 30 minutes. Cell lysates were collected by scraping and debris cleared by centrifugation at 14,000 rpm at 4°C. 50 μl of the lysate was collected as input for total RNA profiling. Lysates were pre-cleared by incubating with 50 μl of protein-G beads (Roche) for 1 hour at 4°C and then collecting supernatants (pre-clearing). Pre-cleared lysates were then incubated with 15 μg of antibody against Argonaute-2 (AGO2)(ab57113, Abcam) at 4°C for 3 hours. AGO2 is a protein component of RISC. Following AGO2 antibody incubation, 50 μl of protein-G beads were added to the lysate and incubated for 1 hour at 4°C. Beads were washed 8 times with lysis buffer and RISC-RNA complexes were extracted by adding 1 mL TRIzol reagent (Invitrogen) directly to the beads. RNA extraction with TRIzol was carried out as per the manufacturers instructions.

### Microarrays

Total RNA or RNA isolated from the RISC immunoprecipitations were amplified and biotin-labeled with the Illumina TotalPrep RNA Amplification Kit (Life Technologies). RNA expression was profiled on HumanHT-12 v4 Expression BeadChips (Illumina) at the KECK Biotechnology Resource Laboratory at Yale University. Microarray data can be found in the GEO repository under accession number GSE63556 (www.ncbi.nlm.nih.gov/geo/).

### RNA-seq Library Preparation and Sequencing

rRNAs were removed using the Ribo-Zero rRNA Removal Kit (Epicentre). The remaining RNA was then fragmented with the NEBNext Magnesium RNA Fragmentation Module (New England BioLabs), and size selected using AMPure XP beads (Agencourt) at 1.8X volume. Ends of the fragmented RNAs were then prepared for adaptor ligation using T4 Polynucleotide Kinase (New England BioLabs). Libraries were prepared from the RNA using the NEBNext Small RNA Library Prep Set according to the manufacturers protocol (New England BioLabs). Libraries were cleaned and further size selected using 0.8X volume AMPure XP beads, and additional size selections with 1.0X beads were performed as necessary. At least 20 million paired end 100bp reads were generated for each replicate using a HiSeq 2000 (Illumina) by the Genomic Sequencing and Analysis Facility at the University of Texas at Austin. RNA-seq data can be found in the GEO repository under accession number GSE63556 (www.ncbi.nlm.nih.gov/geo/).

### RNA-seq Data Processing

Cutadapt was used to trim adaptors, and reads mapping to rRNAs and tRNAs were removed. The remaining reads were mapped to the human genome (hg19) by TopHat2 software version 2.0.9 [25]. Cuffdiff was used to combine biological replicates and assign expression values (FPKM) for each RefSeq gene [25]. To avoid denominator inflation in subsequent ratio calculations, all FPKMs less than 1 were set to 1.

### RNA-seq Gene Expression Repression

Repression of gene expression for each gene was calculated as the ratio of the FPKM in the miR-191 transfection to the FPKM in the control transfection:

$$\begin{array}{c} \text{Repression}=\frac{\text{FPKM for the control }}{\text{FPKM for miR-191}}\\ \; \end{array}$$

### RNA-seq RISC Enrichment

RNA-seq RISC immunoprecipitations (RIP) enrichments for each gene were calculated as follows: RIP FPKMs were first normalized to gene expression FPKMs, and then enrichment was calculated as the ratio of the normalized miR-191 transfection to the normalized control transfection:

$$\text{Enrichment = }\frac{\frac{\text{FPKM for the miR-191 transfection RIP}}{\text{FPKM for the miR-191 transfection gene expression}}}{\frac{\text{FPKM for the control transfection RIP}}{\text{FPKM for the control transfection gene expression}}}$$

### Defining a miRNA Target List using RIP Enrichment and Gene Expression Repression

Each gene was ranked as a putative target by calculating a target score. Target scores were calculated by averaging RIP enrichment values and gene expression repression values. Genes with a score of 1.5 or greater were designated as the target set (S1 Table).

### Gene Ontology Analysis

Gene ontology analysis was performed with the GeneCodis online tool [26–28].

### Luciferase Reporter Assays

Entire 3' UTRs or at minimum 0.5 kb up- and down-stream of the predicted miR-191 binding site (S1 Fig) were cloned from human genomic DNA and inserted into the psi-CHECK2 plasmid (Promega) using XhoI and NotI restriction sites downstream from the *Renilla* luciferase gene. For each plasmid produced this way, a mutant plasmid was subsequently produced via deletion of the 22 bp sequence corresponding to the full length of miR-191 at the putative binding site (QuikChange Lightning Mutagenesis Kit, Agilent). All primers used are listed in S2 Table.

HEK293 cells were seeded and grown overnight in 24-well plates at 10^5^ cells/well in DMEM Supplemented with 10% FBS and 1% Pen-Strep (Hyclone, Gibco). Each plasmid was co-transfected at 50 ng/well with either miR-191 or Control siRNA at a 100 nM in triplicate (Lipofectamine, Invitrogen). Cells were harvested 24 hours post-transfection and luciferase activity was measured using the Promega Dual Luciferase kit according to the manufacturer’s instructions. Data was first normalized per-well by taking the ratio of Renilla luminescence (3’ UTR of interest) to Firefly luminescence (transfection control). The ratio of the mean of the three biological replicates for each miR-191 transfected group to the mean of the corresponding Control siRNA transfected group was calculated as the relative luciferase activity.

### Quantitative Reverse-Transcription PCR

Total RNA from fibroblasts transfected with miR-191 or Control siRNA duplexes was extracted with TRIzol reagent (Invitrogen) and reverse transcribed with the High-Capacity cDNA Reverse Transcription Kit from ABI that uses random hexamers. PCR was performed with SYBR GREEN PCR Master Mix from Applied Biosystems. Target gene transcript levels were normalized to the expression of *GAPDH* and relative mRNA fold changes were calculated by the ΔΔCt method. All primers used are listed in S2 Table, and obtained from qPrimerDepot (primerdepot.nci.nih.gov) [29].

### Western Blotting

Primary human fibroblasts or HeLa cells were seeded in 6-well plates at 5 x 10^4^ cells/well in DMEM Supplemented with 10% FBS (Hyclone, Gibco). 24 hours after plating, miR-191 or Control siRNA duplexes were transfected at a 100 nM concentration (Lipofectamine, Invitrogen). Transfected cells were lysed at 24 hours, 48 hours, 72 hours, or 96 hours post-transfection.

Cell lysates were separated on 4–20% gradient SDS-PAGE gels (Biorad) and proteins transferred onto PVDF membranes. Membranes were blocked with 5% milk or 5% BSA in TBST and probed overnight with primary antibody in blocking solution (AGO2: 1/1000, Abcam, ab57113; CDK9: 1/1000, Abcam, ab76320; NOTCH2: 1/500, ab8926; RPS6KA3: 1/1000, Abcam, ab75832; GAPDH: 1/1000, Abcam, ab9486; Actin: 1/5000, Santa Cruz, sc-10731). Membranes were washed, incubated with HRP-conjugated secondary antibody in blocking solution (1/5000, Santa Cruz Biotechnology, sc-2004), and washed again. HRP substrate solution (Pierce) was added to the membranes and incubated for four minutes, and then blots were exposed to autoradiographic film and developed (Carestream Kodak Biomax). Images of the films were scanned and band intensities quantified using a white-light transilluminator imaging system (FlourChem Q, ProteinSimple). The blot showing reduced expression of miR-191 targets was stripped and re-probed with different primary antibodies for the appropriate proteins.

### Statistical Analysis

As noted, statistical significance was estimated using a one-sided, two sided, or paired Student’s t-test, assuming unequal variance.

## Results

### miR-191 Represses Primary Human Cell Proliferation

**Fig 1:**
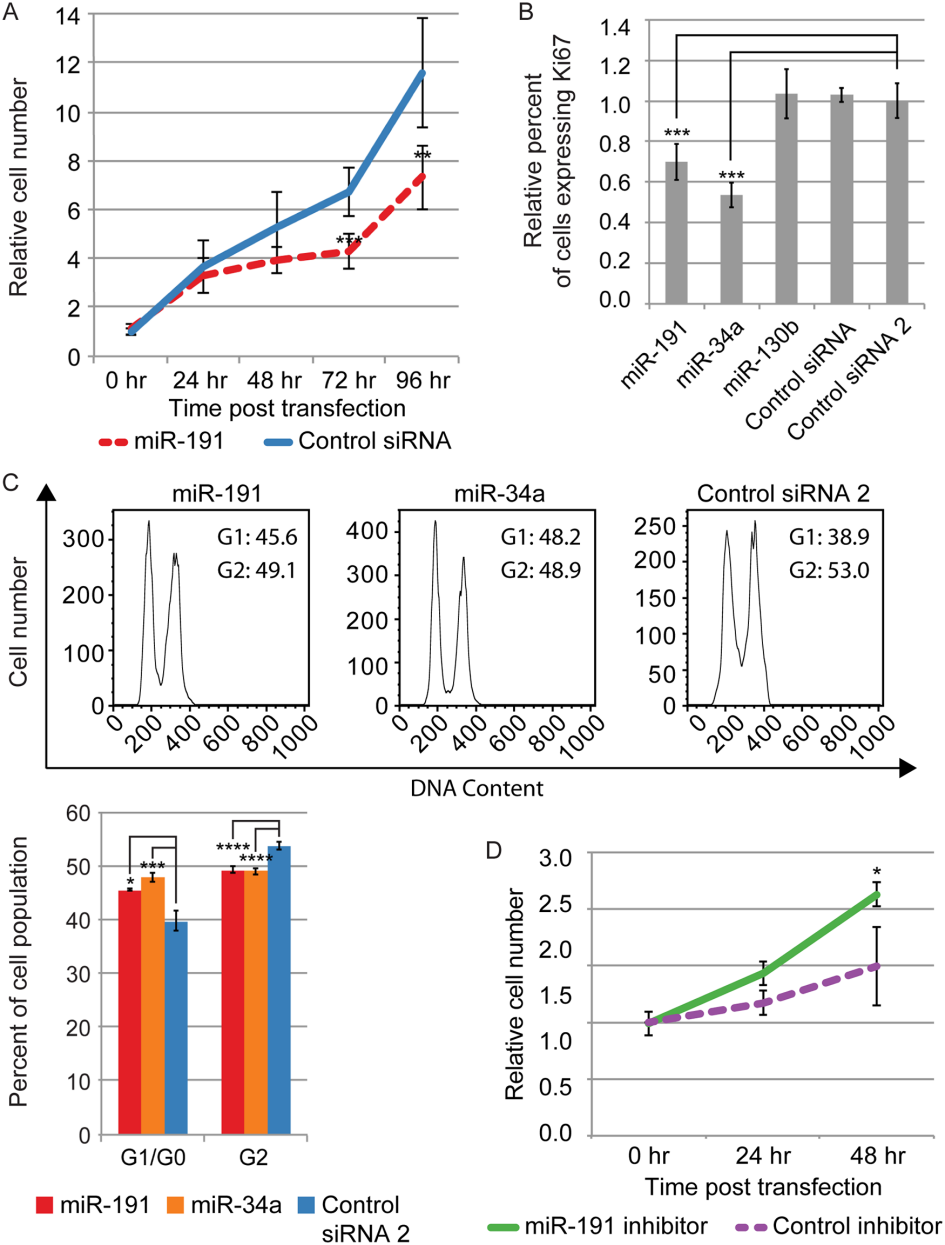
miR-191 represses proliferation. (A) miR-191 transfection reduces the rate of cell growth. Average cell number relative to 0 hr following miR-191 or control siRNA transfection is shown for each time point indicated. Error bars denote ± SD, n = 4 (independent biological replicates). P-values were calculated by Student’s t-test comparing cell numbers following miR-191 transfection to cell numbers following Control siRNA transfection at each time point. (B) miR-191 transfection represses proliferation. The Y-axis indicates the relative percentage of cells expressing Ki67. Bars are the mean percentage of cells expressing Ki67 relative to Control RNA 2, and error bars denote ± SD, n = 3. (C) miR-191 transfection slows progression through the cell cycle. Cell-cycle profiles of transiently transfected fibroblasts following treatment with nocodazole. Representative histograms shown are the median for each treatment of 3 biological replicates. The Y-axis denotes cell number and the X-axis DNA content. Numbers in each histogram indicate percentage of cells in G1 or G2. For the bar graph, the Y-axis denotes the percentage of cells found in each stage of the cell cycle. Error bars indicate ± SD, n = 3. (D) Inhibition of miR-191 increases cell growth in fibroblasts serum starved into quiescence. Average cell number relative to 0 hours post transfection of an LNA targeting miR-191 or a LNA negative control is shown for each time point indicated. Error bars denote ± SD, n = 6. P-values were estimated by Student’s one tailed t-test comparing cell numbers following miR-191 inhibitor transfection to cell numbers following the Control inhibitor transfection at each time point. For B, and C, P-values were calculated by Student’s one tailed t-test. *P < 0.05; **P < 0.01; ***P < 0.001; ****P < 0.0001.

To explore the role of miR-191 in primary human cell proliferation, we transiently overexpressed miR-191 by transfecting the mature duplex form of miR-191 into proliferating primary fibroblasts and assayed cell growth. Transient miR-191 overexpression significantly reduced cell growth compared to control (Fig 1A). In addition, transient miR-191 overexpression significantly reduced the number of cells expressing the proliferation marker Ki67 compared to multiple controls (Fig 1B) [30, 31]. This inhibitory effect of miR-191 was comparable to miR-34a, a well known tumor suppressor [32]. Transient overexpression of miR-191 also significantly decreased the rate of fibroblast progression through the cell cycle (S2 Fig). To more accurately quantify the numbers of cells arrested in G1, we treated cells with the microtubule-destabilizing agent nocodazole, which arrests cells in M phase. Transient miR-191 overexpression in conjunction with M phase trapping confirmed the miR-191 dependent reduction in the rate of progression through the cell cycle (Fig 1C). To rule out indirect effects from flooding the cells and the RNA silencing machinery with large amounts of mature miRNA duplexes, we transiently inhibited miR-191 in fibroblasts induced into quiescence by serum removal. Inhibition of miR-191 in quiescent fibroblasts significantly increased the rate of cell growth (Fig 1D).

### Genome Wide Profiling of miR-191 Targets

**Fig 2:**
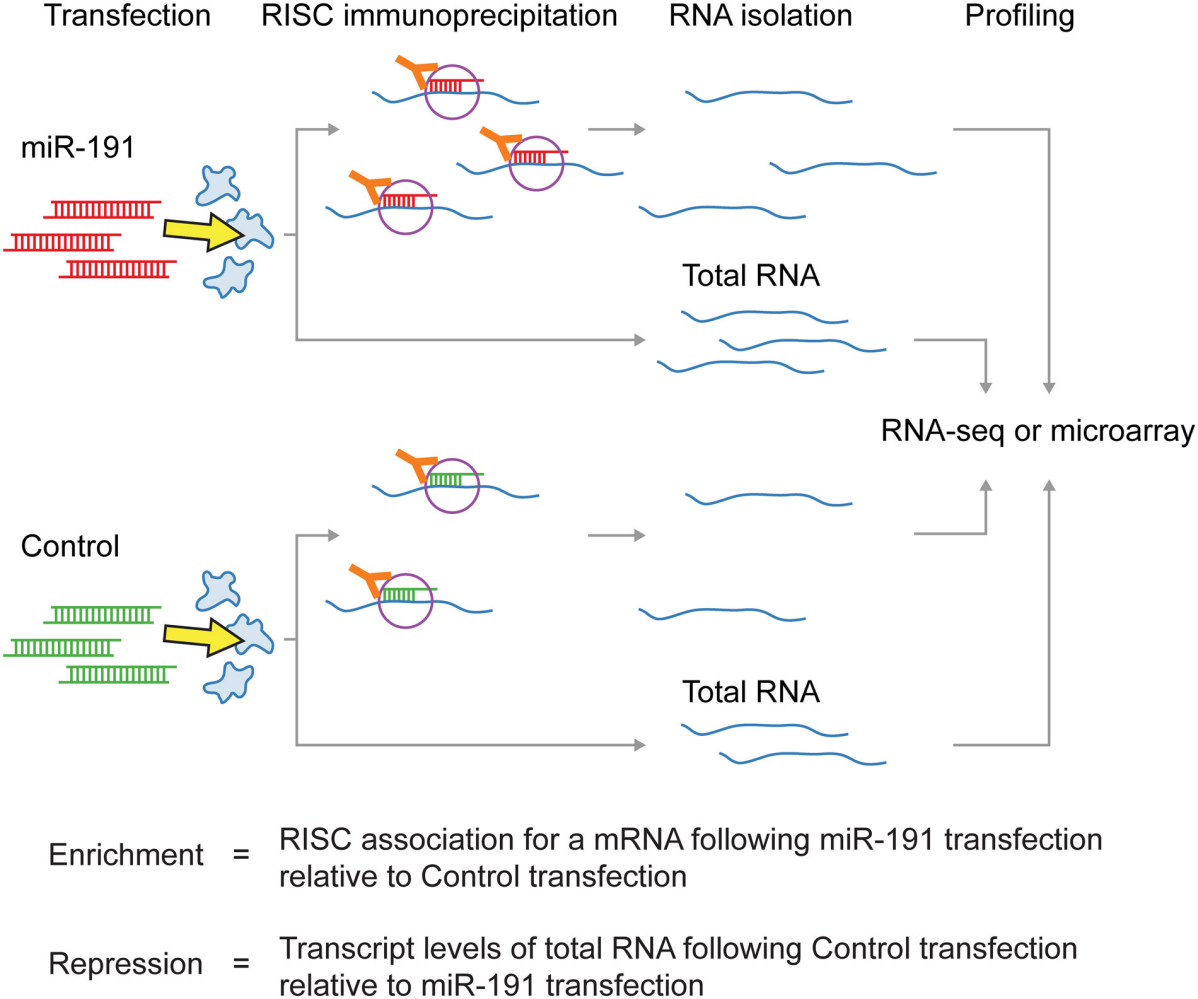
Experimental setup used to profile RISC association and repression of gene expression.

To experimentally identify the targets of miR-191 we used two approaches: (1) Profiling miRNA dependent RISC-transcript association, and (2) profiling miRNA dependent repression of gene expression (Fig 2). RISC is directed to target mRNAs by the mature miRNA guide strand, and mediates repression of gene expression [33]. To immunoprecipitate RISC, we used a monoclonal antibody directed against Argonaute-2, an essential component of RISC [33] (S3 Fig). To profile RISC association, we quantified miRNA dependent enrichment of transcripts isolated from RISC immunoprecipitations (RIPs). We defined RIP enrichment as an increase in RISC association with a given transcript following transient miR-191 transfection compared to the control transfection. Increases in miR-191 dependent RISC association with a given transcript as measured by RIP enrichment were used to identify direct targets of miR-191. We profiled RISC association by quantifying transcripts isolated from RIPs using both microarrays (RIP-ChIP) and RNA-seq (RIP-seq), and gene expression also using both microarrays and RNA-seq. RNA-seq and microarray experiments correlated well (S3 Table). To obtain enough RNA for successful genome wide profiling, we performed all RIPs and gene expression experiments in HeLa cells, due to HeLa cells having a greater RNA content and being of a smaller size than human fibroblasts.

**Fig 3:**
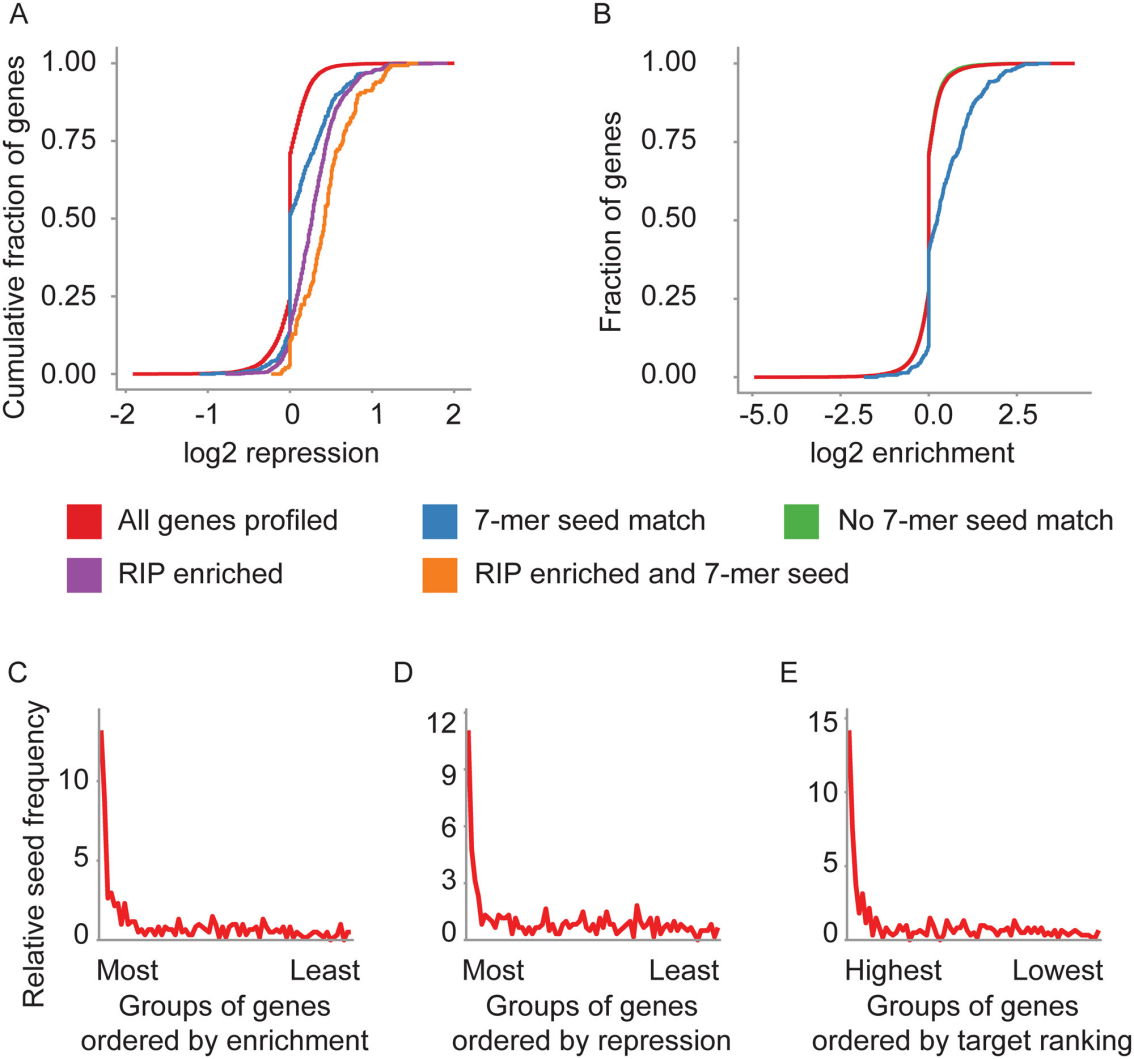
Genome wide profiling of miR-191 targets. (A) Transcript levels of mRNAs enriched in the RIP-seq following miR-191 transfection were decreased in the RNA-seq expression profiles. The Y-axis indicates the cumulative fraction of mRNAs profiled for each group of mRNAs denoted by line color, and the X-axis shows the level of repression for each mRNA profiled with positive values indicating increased repression. mRNAs that contain a miR-191 miRNA 7-mer seed match were significantly more repressed than all mRNAs profiled (p = 4.65e-24). mRNAs enriched in the RIP were significantly more repressed than mRNAs with a miR-191 7-mer seed match (p = 2.54e-12). mRNAs enriched in the RIP that contain a 7-mer miR-191 seed match were significantly more repressed than mRNAs enriched in the RIP (p = 3.97e-17) Significance estimates were calculated with Student’s t-test. (B) mRNAs with a miR-191 seed match were significantly enriched in the RIP-seq compared to all mRNAs profiled (p = 4.65e-24, Student’s t-test). The Y-axis shows the cumulative fraction of mRNAs profiled for each group of mRNAs denoted by line color, and the X-axis indicates the amount of enrichment in the RIP with positive values indicating increased enrichment. (C) mRNAs most enriched in the RIP-seq had the highest frequency of miR-191 seed matches. The X-axis denotes consecutive groups of 250 genes, ranked from most enriched to least enriched in the RIP-seq. The line is the frequency indicated on the Y-axis of the miR-191 7-mer seed match in the 3’ UTRs of the group relative to the frequency of the seed match in all mRNAs profiled. (D) mRNAs repressed in the gene expression experiments had the highest frequency of miR-191 7-mer seed matches. The X-axis indicates consecutive groups of 250 genes, ranked from most repressed to least repressed. The line is the frequency indicated on the Y-axis of miR-191 7-mer seed matches in the 3’ UTRs of the group relative to the frequency of miR-191 seed matches in all mRNAs profiled. (E) The highest ranked miR-191 targets had the highest frequency of miR-191 seed matches. RIP-seq enrichment and gene expression repression data were combined to rank miR-191 targets. The X-axis denotes consecutive groups of 250 genes, from most highly ranked to least highly ranked. The line is the frequency indicated on the Y-axis of miR-191 7-mer seed matches in the 3’ UTRs of the group relative to the frequency of seed matches in all mRNAs profiled. For A-D, n = 3. E combines gene expression RNA-seq data, n = 3, with RIP-seq data, n = 3.

To assess the effectiveness of our profiling of miR-191 targets, we first examined the levels of repression for mRNAs that associated with RISC in a miR-191 dependent manner. miR-191 dependent RISC association correlated well with repression of expression (Fig A in S4 Fig), and mRNAs that associated with RISC in a miR-191 dependent manner were significantly more repressed than all mRNAs profiled (Fig 3A). In addition, transcript levels of mRNAs that associated with RISC in a miR-191 dependent manner were significantly more repressed than all mRNAs profiled that contained a miR-191 7-mer seed match in their 3’ UTR, indicating that the RIP more successfully identifies mRNAs repressed by miR-191 transient overexpression than using presence of the seed match as the lone criteria (Fig 3A). mRNAs that were both RISC associated and contained a miR-191 7-mer seed match showed the greatest amount of repression (Fig 3A).

As an additional means to assess the quality of our miR-191 target profiles, we quantified miR-191 seed match frequency in miR-191 dependent RISC associated mRNAs. mRNAs that contained a 7-mer miR-191 seed match in their 3’ UTR displayed significantly higher levels of miR-191 dependent RISC association than all mRNAs profiled (Fig 3B). Conversely, mRNAs with the greatest miR-191 dependent RISC association had the highest frequency of miR-191 seed sites in their 3’ UTRs (Fig 3C), and mRNAs with the greatest miR-191 dependent repression of gene expression had the highest frequency of miR-191 seed sites in their 3’ UTRs (Fig 3D). Using both miR-191 dependent RISC association and repression of gene expression to identify putative miR-191 targets resulted in the highest frequency of seed sites in the most highly ranked targets (Fig 3E). In addition, enrichment of sequences pairing to miR-191 in the most highly ranked targets was specific to sequences matching the seed area of miR-191, and not subsequences pairing to other areas of the miRNA (Fig B in S4 Fig).

### mRNAs with miR-191 Seed Matches in Their Coding Sequence Display Greater RISC Association and Repression of Gene Expression than mRNAs with Seed Matches in Their 3’ UTR

**Fig 4:**
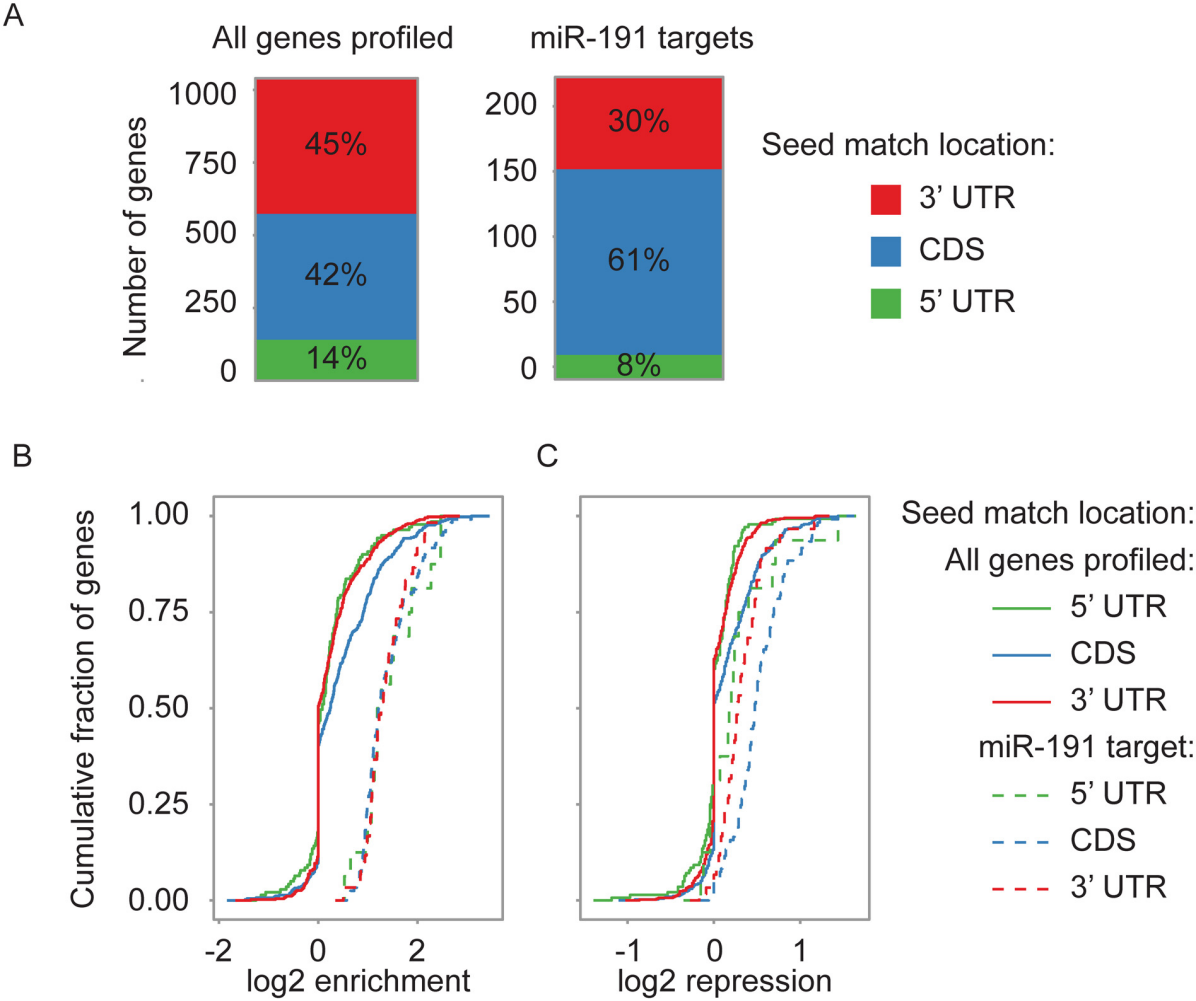
Greater RISC association and repression of mRNAs with a miR-191 seed match in the CDS than in the 3’ UTR. (A) RIP-seq enriched mRNAs have a higher proportion of miR-191 seed matches in the CDS than all mRNAs profiled. Left panel: All mRNAs profiled with a 7-mer miR-191 seed match. Right panel: mRNAs 1.5 fold enriched in the RIP-seq with a 7-mer miR-191 seed match. The Y-axis indicates the number of genes, and boxes show the proportion of genes with a seed match in the location indicated by color. (B) mRNAs with a miR-191 seed match in the CDS were significantly more enriched in the RIP-seq than mRNAs with a miR-191 seed match in the 3x UTR or 5’ UTR (p = 1.84e-06 and p = 6.50e-05 respectively). The X-axis shows the enrichment level in the RIP-seq. (C) mRNAs with a miR-191 seed match in the CDS were significantly more repressed in the RNA-seq gene expression profiling than mRNAs with a seed match in the 3’ UTR or 5’ UTR (p = 6.35e-10 and p = 8.87e-08, respectively for all genes profiled; and p = 1.17e-05 and p = 0.02 respectively for the set of miR-191 target genes). The X-axis shows amount of repression measured by RNA-seq. (B and C) The cumulative fraction of all mRNAs profiled with the indicated seed match location is shown on the Y-axis. Colors denote the seed match location. Solid lines are all mRNAs profiled with a miR-191 seed match, and dashed lines are the mRNAs 1.5 fold enriched or repressed with a seed match. For A, B, and C, n = 3. For B and C, P-values were estimated by Student’s t-test.

Profiling miRNA dependent changes in gene expression has shown that efficacious miRNA-target pairing occurs through the 3’ UTR [12, 34]. Extensive RISC association with the CDS (coding sequence) mediated by a subset of miRNAs has been detected, but repression of gene expression mediated by CDS targeting was minor [35–39]. This implies that although there is RISC occupancy of the CDS, functional targeting occurs through the 3’ UTR. Due to a large fraction of miR-191 7-mer seed matches being located in the CDS of protein coding genes, we hypothesized that miR-191 may mediate extensive RISC occupancy of the CDS, but exert a stronger influence on gene expression through 3’ UTR pairing (Fig 4A). In support of this hypothesis, we observed an increase in the proportion of miR-191 seed matches in the CDS of our experimentally identified miR-191 mRNA target set compared to all mRNAs profiled (61% compared to 42%) (Fig 4A and Fig A in S5 Fig). In addition, mRNAs with a miR-191 seed match in the CDS showed a significant increase in miR-191 dependent RISC association compared to mRNAs with a seed match in the 5’ UTR or 3’ UTR (Fig 4B). For mRNAs with the greatest miR-191 dependent RISC association, there were no consistently significant differences in RISC association between mRNAs with seed matches in the 3’ UTR, CDS, or 5’ UTR (Fig 4B and Fig B in S5 Fig). Surprisingly, miR-191 dependent repression of gene expression was significantly greater when a seed match was located in the CDS compared to the 3’ UTR (Fig 4C and Fig C in S5 Fig). Taken together, these results suggest that miR-191 may mediate extensive RISC occupancy of the CDS, and exert a greater effect on gene expression through pairing to the CDS than the 3’ UTR.

### miR-191 Directly Targets Multiple Proto-Oncogenes

**Fig 5:**
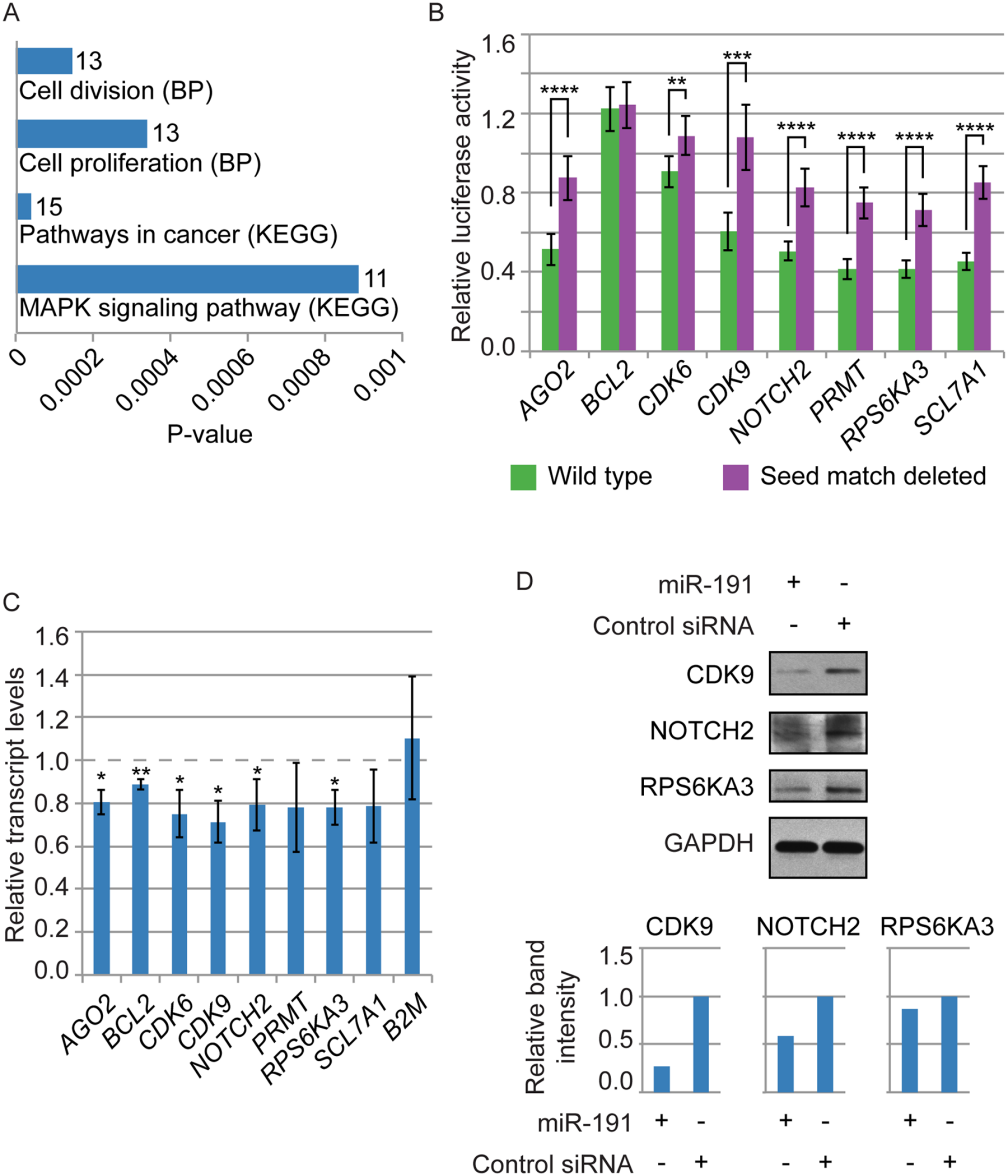
miR-191 directly targets multiple proto-oncogenes. (A) Enriched Gene Ontology terms for the experimentally identified set of miR-191 targets. BP: Biological process. Numbers indicate gene counts. (B) Luciferase reporter assays showed miR-191 directly targets the 3’ UTRs of the genes indicated on the X-axis. The Y-axis denotes relative luciferase units from miR-191 transfected HEK293 cells normalized to Control siRNA transfected cells. Purple bars: 3’ UTRs with the putative miR-191 target site entirely deleted. Green bars: Intact 3’ UTRs. P-values were estimated by Student’s one tailed t-test comparing miR-191 normalized to Control siRNA luciferase activity with intact 3’ UTRs to normalized luciferase activity with miR-191 target site deleted 3’ UTRs. (C) miR-191 transfection significantly decreased *AGO2*, *BCL2*, *CDK6*, *CDK9*, *NOTCH2*, and *RPS6KA3* transcript levels in fibroblasts. Fold changes are indicated on the Y-axis relative to the Control siRNA transfection. GAPDH was used to normalize input RNA levels. P-values were estimated by Student’s one tailed t-test comparing normalized transcript levels following miR-191 transfection to normalized transcript levels following Control siRNA transfection. (D) miR-191 transfection in fibroblasts decreased protein expression of CDK9, NOTCH2, and RPS6KA3 compared to Control siRNA transfection. Band intensities were quantified, normalized to GAPDH, and shown relative to the Control siRNA. For B and C, bars indicate the mean, and error bars denote ± SD, n = 6 and 3 respectively. *P < 0.05; **P < 0.01; ***P < 0.001; ****P < 0.0001.

In our experimentally identified set of miR-191 targets, there was strong enrichment for genes associated with cell proliferation, cell division, the MAPK signaling pathway, and cancer pathways (Fig 5A). Because we had found the primary phenotypic effect of miR-191 overexpression to be inhibition of proliferation, we selected multiple proto-oncogenes and regulators of proliferation to further investigate as miR-191 targets. To confirm direct miR-191 targeting and regulation of the transcripts produced from this group of genes, we cloned ~0.5–1 kb sections of their 3’ UTRs into luciferase reporter constructs. CDK6 was one of our putative targets and had previously been identified as a direct target of miR-191 by multiple groups [19, 21, 40]. The luciferase reporter assays confirmed 7 of the 8 putative targets as direct targets of miR-191, and deletions of the miR-191 seed matches showed that the miR-191 mediated repression was dependent on the miR-191 seed matches in the 3’ UTRs (Fig 5B). To further confirm these genes as direct targets and examine the effect of miR-191 on the expression of these genes in primary human fibroblasts, we examined the effect of miR-191 transient overexpression in primary fibroblasts and HeLa cells on transcript and protein levels. Transient miR-191 overexpression in fibroblasts significantly repressed the transcript levels of *AGO2*, *BCL2*, *CDK6*, *CDK9*, *NOTCH2*, and *RPS6KA3*, but not *PRMT* or *SLC7A1* (p = 0.11 and p = 0.08, respectively) (Fig 5C). We assayed the effect of transient miR-191 overexpression on protein levels in fibroblasts and HeLa cells for a subset of the genes, and confirmed miR-191 repression of CDK9, NOTCH2, and RPS6KA3, but AGO2 only showed repression in HeLa cells (Fig 5D, S6 Fig, and data not shown). This discrepancy may be due to differences in protein stability and transfection efficiency, in combination with the transient nature of the overexpression. To examine the effect of miRNA concentration on miR-191 mediated repression, we conducted luciferase reporter assays with decreasing concentrations of miRNA transfected for this subset of the confirmed miR-191 targets, and observed similar miRNA mediated repression at lower concentrations (S7 Fig).

## Discussion

miRNA target prediction algorithms are in general developed from experimentally determined mechanisms of miRNA target pairing, but our understanding of miRNA targeting mechanisms is still incomplete. In this study, we show that miR-191 regulates proliferation in primary human fibroblasts and show by experimental analysis that it targets a number of proto-oncogenes. Constructing genome-wide target profiles as we have done in this work, aids in refining our understanding of miRNA target pairing rules and in improving prediction algorithms. For instance, miRNA target pairing has been generally thought to occur through pairing to the 3’ UTR of the target transcript, and prediction algorithms frequently restrict predictions to the 3’ UTR [33], but in this work we show that miR-191 may utilize extensive pairing to the CDS of the target transcript (Fig 4). By constructing a genome wide miR-191 target set, we were able identify and confirm the regulators of proliferation CDK9, NOTCH2, and RPS6KA3 as direct targets of miR-191. We demonstrated that miR-191 represses proliferation and cell cycle progression in primary human fibroblasts. In addition, our results suggest that miR-191 mediates gene expression of a large fraction of its target genes by targeting RISC to the CDS.

CDK9 regulates RNA polymerase II controlled gene expression to mediate cell growth, proliferation, apoptosis and differentiation [41]. CDK9 activity is deregulated in numerous malignancies and human pathologies, and is a target for pharmacological inhibition for cancer therapies [41]. NOTCH2 is overexpressed in multiple cancer types and has been shown to have oncogenic effects, including on proliferation [42, 43]. RPKS6KA3 is part of the MAPK3 pathway, and promotes proliferation in multiple cancer types [44, 45]. Although miR-191 regulation of CDK9, NOTCH2, and RPS6KA3 expression was modest, moderate repression of multiple key regulators of proliferation may have large phenotypic effects in concert.

In addition to the novel miR-191 targets we identified in this study, miR-191 has been previously shown to target the proliferation associated genes NDST1 in the human gastric carcinoma cell line MGC803, and CDK6, SATB1, CCND2, CSDA, and EGR1 in the human embryonic kidney cell line used in this study HEK293 [18, 21, 46]. A miR-191 dependent repression of protein level was shown for NDST1 in MGC803s, CDK6 and SATB1 in human epidermal keratinocytes, and CCND2, CSDA, and EGR1 in the human breast cancer cell line MDA-MB-231 [18, 21, 46]. This collection of experimentally validated proliferation associated miR-191 targets raises the intriguing possibility that miR-191 may regulate proliferation by targeting a network of genes connected to proliferative pathways, rather than exerting its effects through modulation of one or two genes. Several of the previously identified miR-191 targets, such as NDST1, SATB1, and EGR1 are more strongly associated with inhibition of proliferation, where as CSDA, CCND2, CDK6 are known to promote proliferation, in addition to the targets identified in this study, CDK9, NOTCH2, and RPKS6KA3 [18, 21, 46]. The direct targeting of both proliferation enhancing and inhibiting genes is consistent with observations of miR-191 functioning as a tumor suppressor in certain cell types or genetic backgrounds and an oncogene in other contexts.

As may be expected given the function of miR-191 as a regulator of proliferation, it is unsurprising that miR-191 expression is frequently altered in tumors, and may be up or down regulated depending on cancer type [40]. miR-191 has predominantly been shown to act as an oncogene, promoting tumorigenesis in gastric, colorectal, breast, thyroid, and hepatocellular carcinoma, but has also been shown to inhibit tumorigenesis in thyroid carcinoma and breast cancer [16, 19, 20, 40]. In multiple cases, miR-191 influences tumor progression by regulating proliferation; miR-191 promotes proliferation in hepatocellular carcinoma, and in gastric carcinoma by targeting NDST1, but inhibits proliferation in thyroid carcinoma by targeting CDK6 [18, 20]. The duality of miR-191 is illustrated in breast cancer: miR-191 impairs or promotes breast cancer tumorigenesis depending on ER-alpha receptor status [19, 46]. Taken as a whole, the cell type, cancer, and cancer subtype specific effects of miR-191 on proliferation suggest miR-191 regulates proliferation in a manner dependent upon cell type and genetic context.

Gene expression profiling following miRNA perturbation has shown that miRNA seed matches in the CDS have little effect on transcript levels [12, 34]. Genome wide profiling of miRNA dependent RISC-transcript association has revealed extensive RISC occupancy of the CDS, but RISC occupancy of the CDS had generally negligible effects on transcript levels [35–39]. At most, miRNA target pairing sites in the CDS exert about half as strong of an effect on transcript levels as target pairing sites in the 3’ UTR [36]. We observed a large fraction of miR-191 seed matches in the CDS, and extensive miR-191 dependent RISC occupancy of the CDS. However, we also showed extensive miR-191 dependent regulation of transcript levels for mRNAs with a miR-191 seed match in the CDS, and the mRNAs with a seed match in their CDS showed a significantly stronger response than those with a 3’ UTR seed match. The extensive miR-191 mediated regulation of gene expression through seed matches in the CDS may represent an isolated case, but it raises the intriguing possibility that other miRNAs may significantly regulate gene expression through pairing to target sites in the CDS.

## Supporting Information

S1 Fig Diagram of miR-191 pairing to putative targets. Numbers in parenthesis indicate location in the 3’ UTR, not genomic coordinates. Vertical lines show base pairing.
(TIF)

S2 Fig miR-191 transfection slows progression through the cell cycle. Cell-cycle profiles of transiently transfected fibroblasts were measured as shown in Fig 1C. The Y-axis denotes the mean percentage of cells found in each stage of the cell cycle. Error bars indicate ± SD, n = 3. P-values were estimated by Student’s t-test. *P < 0.05; **P < 0.01; ***P < 0.001.
(TIF)

S3 Fig Ago2 is pulled down by RISC immunoprecipitation. Western blot using the antibody directed against Ago2 of the RISC immunoprecipitation sample and mock immunoprecipitation sample where mouse serum was used instead of Ago2 antibody. Pre IP: Sample from cell lysates collected prior to immunoprecipitation; Supernatant: Supernatant removed from immunoprecipitation; IP: Sample recovered after immunoprecipitation. Actin was used as a loading control.
(TIF)

S4 Fig Correlation of target association and repression. (A) Comparison of enrichment in the RIP-seq to repression of gene expression for each mRNA profiled. Pearson correlation = 0.66. The Y-axis shows log2 transformed RIP-seq enrichment, and the X-axis log2 transformed repression of gene expression assayed by RNA-seq. n = 3. (B) There is a high frequency of sequences pairing only to the seed region of miR-191 in the 3’ UTRs of the miR-191 target set. The frequency of 6-mer sequences pairing to each 6-mer sub-sequence of miR-191 is plotted. Bars are the frequency denoted on the Y-axis of a 6-mer in the 3’ UTRs of the experimentally identified miR-191 target set relative to all mRNAs profiled. 6-mers are organized along the X-axis from 5’ end to 3’ end of the mature miRNA.
(TIF)

S5 Fig Microarray data confirms greater RISC association and repression of mRNAs with a miR-191 seed match in the CDS than in the 3’ UTR. (A) RIP-ChIP enriched mRNAs have a higher proportion of miR-191 seed matches in the CDS than all mRNAs profiled. Left panel: All mRNAs profiled with a 7-mer miR-191 seed match. Right panel: mRNAs 1.5 fold enriched in the RIP-ChIP with a 7-mer miR-191 seed match. The Y-axis indicates the number of genes, and boxes show the number of genes with a seed match in the location indicated by color. (B) mRNAs with a miR-191 seed match in the 3’ UTR were significantly more enriched than mRNAs with a miR-191 seed match in the CDS (p = 3.89e-03). The X-axis shows enrichment level in the RIP-ChIP. (C) mRNAs with a miR-191 seed match in the CDS were significantly more repressed in the microarray gene expression profiling than mRNAs with a seed match in the 3’ UTR (p = 3.93e-05) for all genes profiled or in the 3’ UTR or 5’ UTR for the set of miR-191 target genes (p = 8.70e-4 and p = 2.35e-3, respectively). The X-axis shows amount of repression measured by microarrays. (B and C) The cumulative fraction of all mRNAs profiled with the indicated seed match location is shown on the Y-axis. Colors denote the seed match location. Solid lines are all mRNAs profiled with a miR-191 seed match, and dashed lines are the mRNAs 1.5 fold repressed or enriched with a seed match. For A, B, and C, n = 3. For B and C, P-values were estimated by Student’s t-test.
(TIF)

S6 Fig miR-191 transfection in HeLa cells decreased protein expression of CDK9, NOTCH2, RPS6KA3, and AGO2 compared to Control siRNA transfection. Band intensities were quantified, normalized to GAPDH, and shown relative to the Control siRNA.
(TIF)

S7 Fig miR-191 directly targets the 3’ UTRs of multiple proto-oncogenes. Luciferase reporter assays showed miR-191 directly targets the 3’ UTRs of the genes indicated on the X-axis. Varying concentrations of miR-191 were used, and final concentrations of miR-191 are indicated on the X-axis. The Y-axis denotes relative luciferase units from miR-191 transfected HEK293 cells normalized to Control siRNA transfected cells. Purple bars: 3’ UTRs with the putative miR-191 target site deleted. Green bars: Intact 3’ UTRs. Bars indicate the mean, and error bars denote ± SD, n = 3. P-values were estimated by Student’s one tailed t-test comparing miR-191 normalized to Control siRNA luciferase activity with intact 3’ UTRs to luciferase activity with miR-191 target site deleted 3’ UTRs. *P < 0.05; **P < 0.01; ***P < 0.001; ****P < 0.0001.
(TIF)

S1 Table Experimentally identified targets of miR-191. (DOCX)

S2 Table Primers used for qRT-PCR, cloning, and mutagenesis. (DOCX)

S3 Table Correlation between RNA-seq and microarrays. Spearman rank correlation between RNA-seq FPKM and microarray intensity value for each gene profiled by both RNA-seq and FPKM. For all conditions, the FPKM and microarray intensity values of the biological replicates were averaged for each gene profiled. Values found in the table are the Spearman rank correlation coefficients calculated comparing the averaged RNA-seq FPKM and microarray intensity values.
(DOCX)
