# Supplementary figures and images for: MiR-191 Regulates Primary Human Fibroblast Proliferation and Directly Targets Multiple Oncogenes
Source: PLoS One. 2015 May 20;10(5):e0126535. doi: 10.1371/journal.pone.0126535 (PMC4439112; doi:10.1371/journal.pone.0126535)

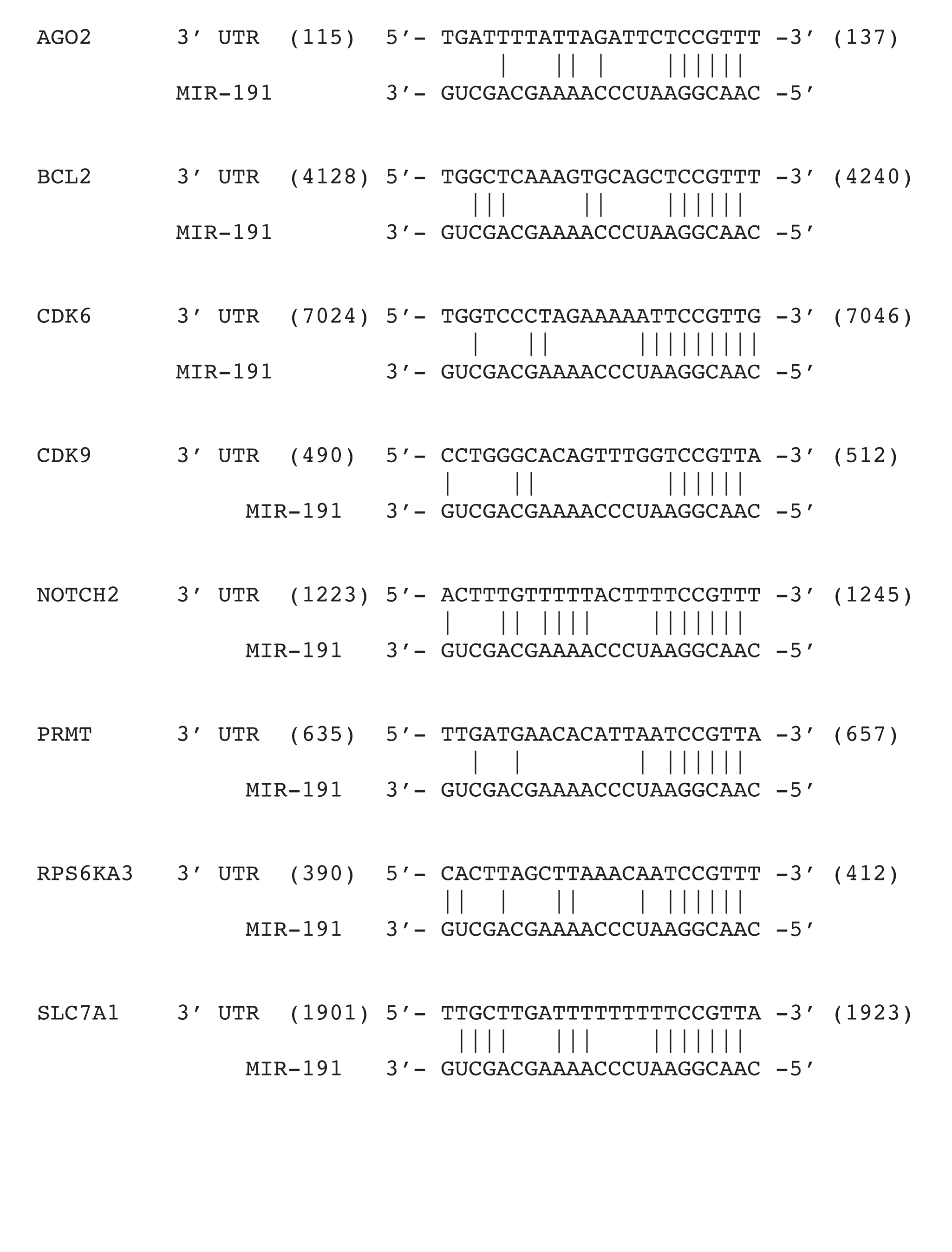

Supplement: S1 Fig — Numbers in parenthesis indicate location in the 3’ UTR, not genomic coordinates. Vertical lines show base pairing. (TIF) [file pone.0126535.s001.tif]

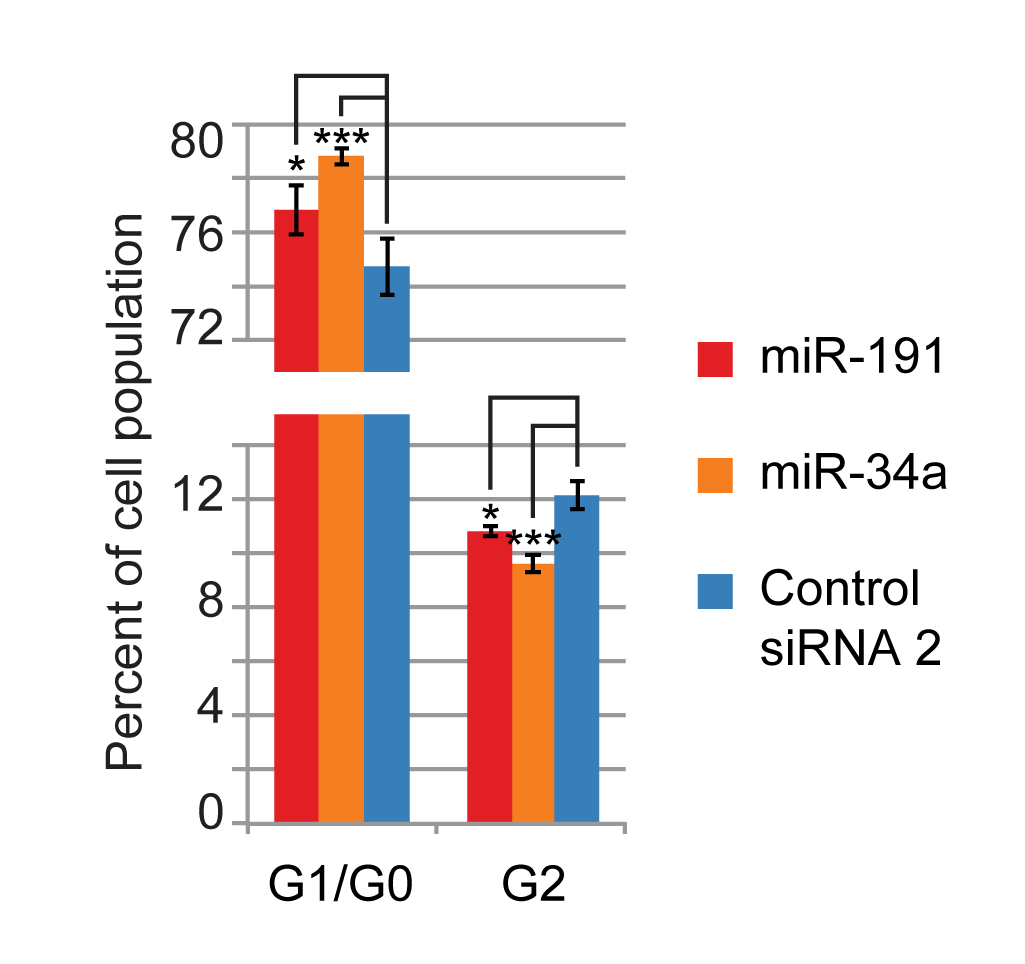

Supplement: S2 Fig — Cell-cycle profiles of transiently transfected fibroblasts were measured as shown in Fig 1C. The Y-axis denotes the mean percentage of cells found in each stage of the cell cycle. Error bars indicate ± SD, n = 3. P-values were estimated by Student’s t-test. *P < 0.05; **P < 0.01; ***P < 0.001. (TIF) [file pone.0126535.s002.tif]

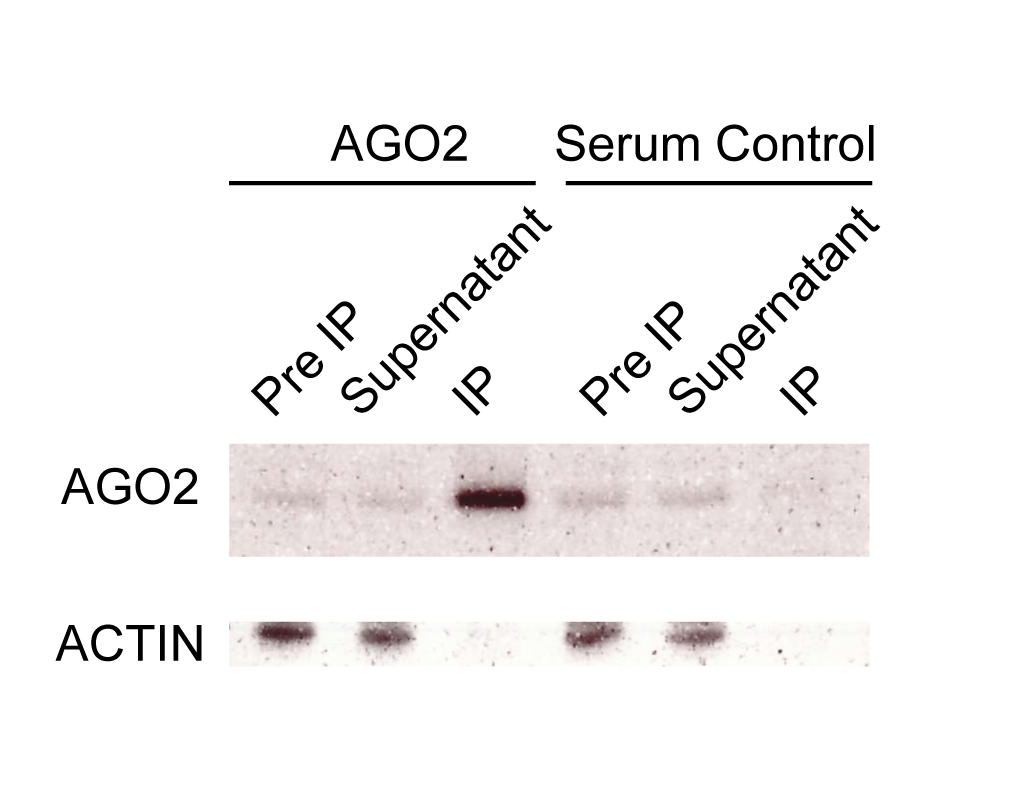

Supplement: S3 Fig — Western blot using the antibody directed against Ago2 of the RISC immunoprecipitation sample and mock immunoprecipitation sample where mouse serum was used instead of Ago2 antibody. Pre IP: Sample from cell lysates collected prior to immunoprecipitation; Supernatant: Supernatant removed from immunoprecipitation; IP: Sample recovered after immunoprecipitation. Actin was used as a loading control. (TIF) [file pone.0126535.s003.tif]

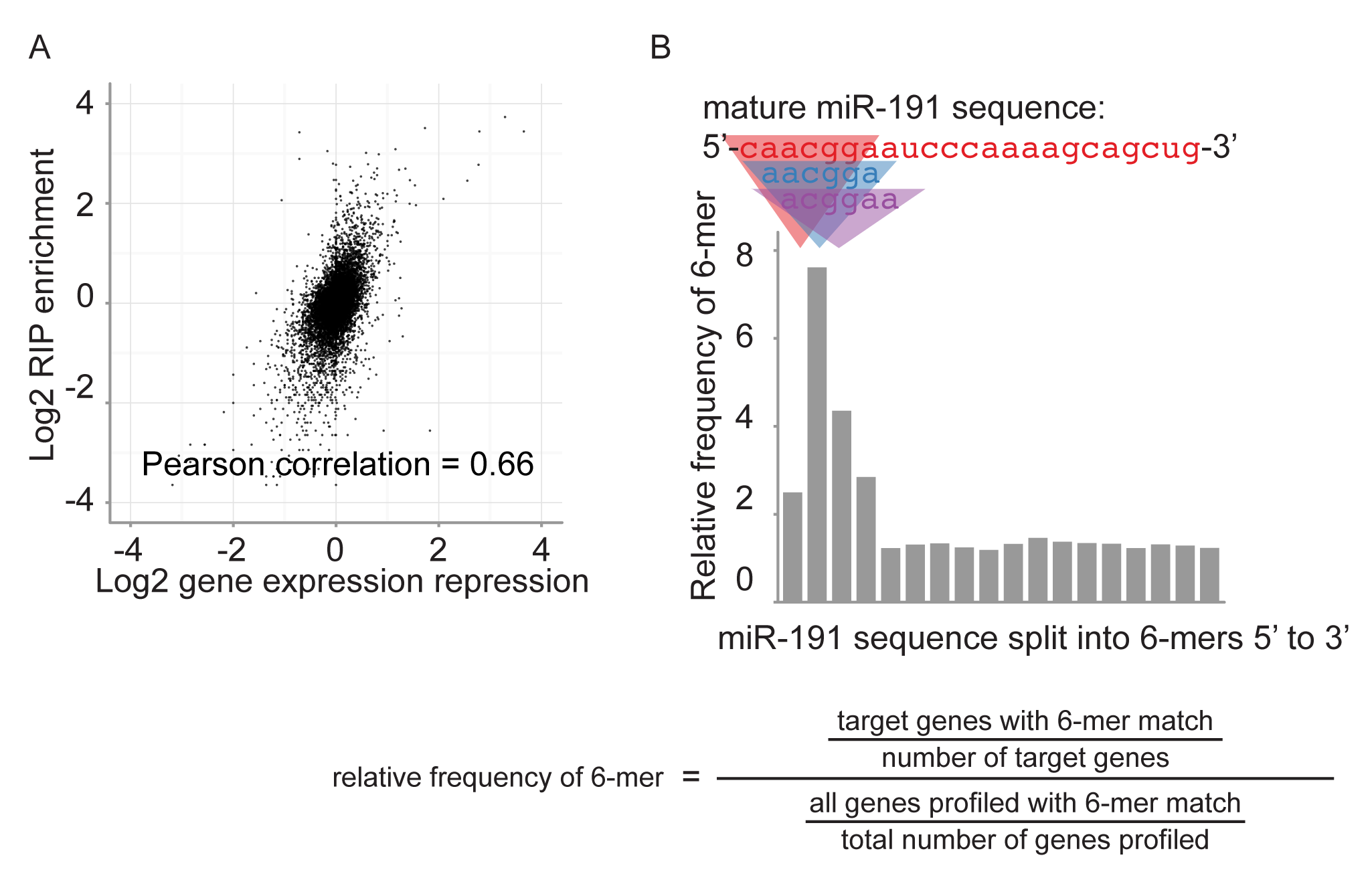

Supplement: S4 Fig — (A) Comparison of enrichment in the RIP-seq to repression of gene expression for each mRNA profiled. Pearson correlation = 0.66. The Y-axis shows log2 transformed RIP-seq enrichment, and the X-axis log2 transformed repression of gene expression assayed by RNA-seq. n = 3. (B) There is a high frequency of sequences pairing only to the seed region of miR-191 in the 3’ UTRs of the miR-191 target set. The frequency of 6-mer sequences pairing to each 6-mer sub-sequence of miR-191 is plotted. Bars are the frequency denoted on the Y-axis of a 6-mer in the 3’ UTRs of the experimentally identified miR-191 target set relative to all mRNAs profiled. 6-mers are organized along the X-axis from 5’ end to 3’ end of the mature miRNA. (TIF) [file pone.0126535.s004.tif]

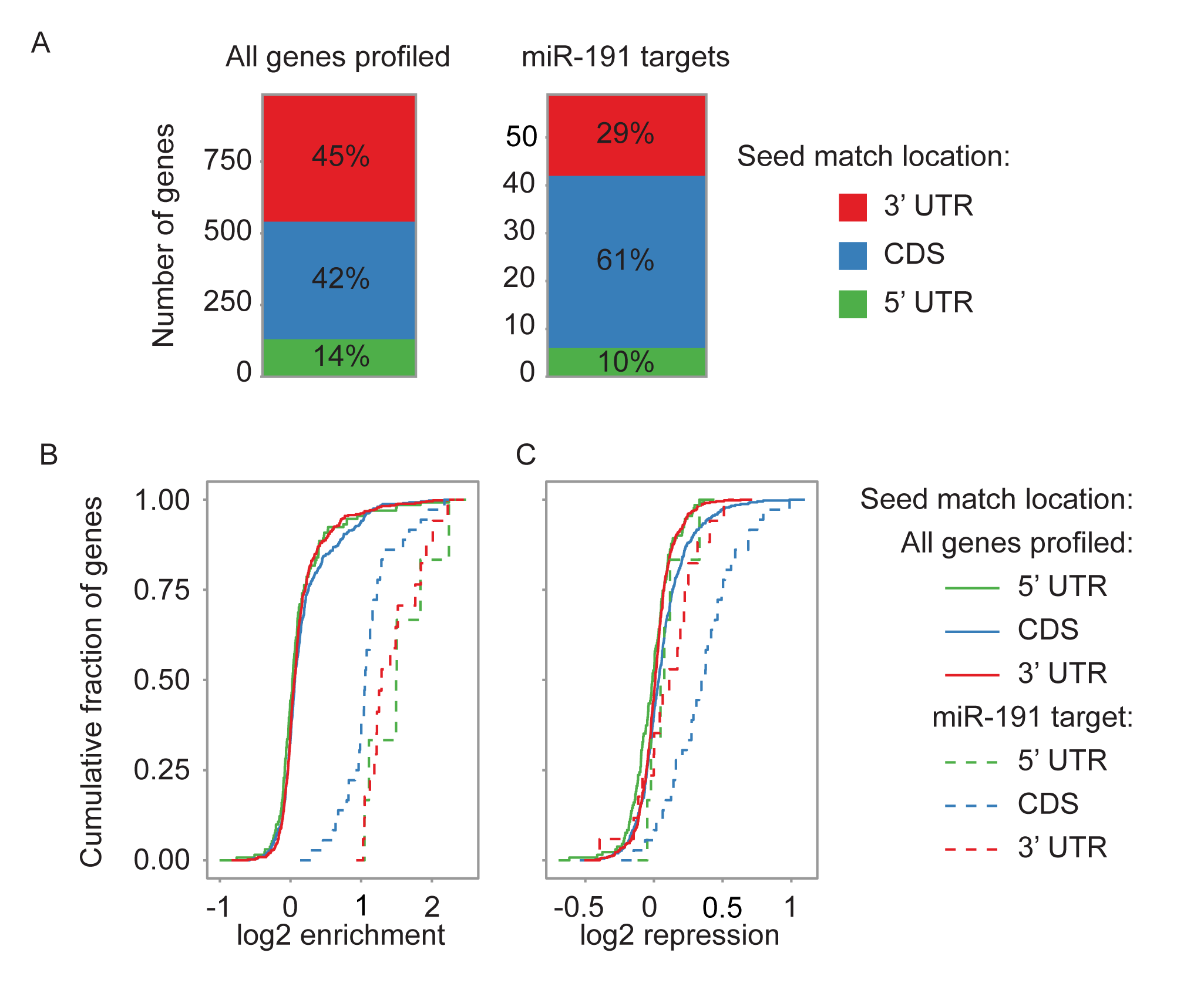

Supplement: S5 Fig — (A) RIP-ChIP enriched mRNAs have a higher proportion of miR-191 seed matches in the CDS than all mRNAs profiled. Left panel: All mRNAs profiled with a 7-mer miR-191 seed match. Right panel: mRNAs 1.5 fold enriched in the RIP-ChIP with a 7-mer miR-191 seed match. The Y-axis indicates the number of genes, and boxes show the number of genes with a seed match in the location indicated by color. (B) mRNAs with a miR-191 seed match in the 3’ UTR were significantly more enriched than mRNAs with a miR-191 seed match in the CDS (p = 3.89e-03). The X-axis shows enrichment level in the RIP-ChIP. (C) mRNAs with a miR-191 seed match in the CDS were significantly more repressed in the microarray gene expression profiling than mRNAs with a seed match in the 3’ UTR (p = 3.93e-05) for all genes profiled or in the 3’ UTR or 5’ UTR for the set of miR-191 target genes (p = 8.70e-4 and p = 2.35e-3, respectively). The X-axis shows amount of repression measured by microarrays. (B and C) The cumulative fraction of all mRNAs profiled with the indicated seed match location is shown on the Y-axis. Colors denote the seed match location. Solid lines are all mRNAs profiled with a miR-191 seed match, and dashed lines are the mRNAs 1.5 fold repressed or enriched with a seed match. For A, B, and C, n = 3. For B and C, P-values were estimated by Student’s t-test. (TIF) [file pone.0126535.s005.tif]

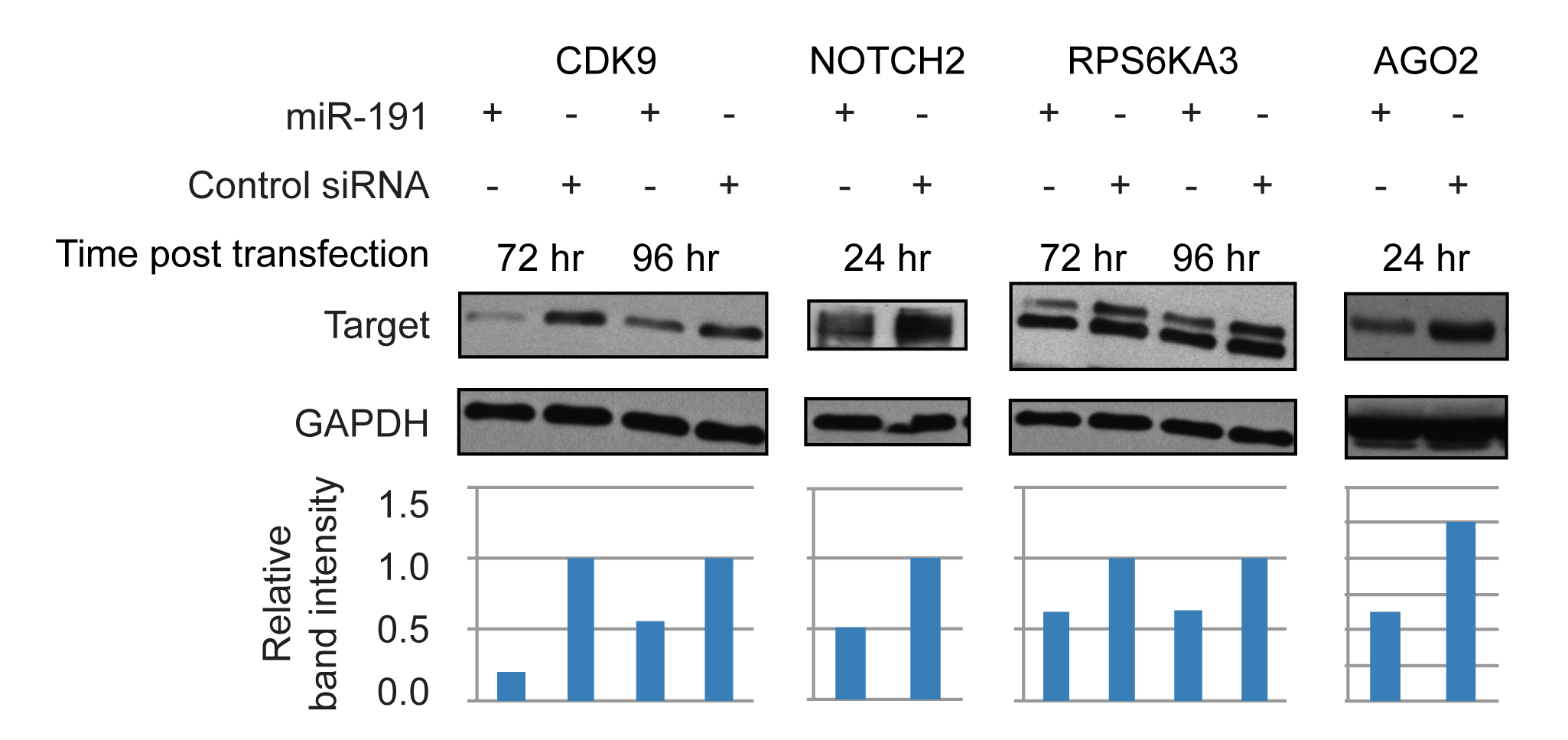

Supplement: S6 Fig — Band intensities were quantified, normalized to GAPDH, and shown relative to the Control siRNA. (TIF) [file pone.0126535.s006.tif]

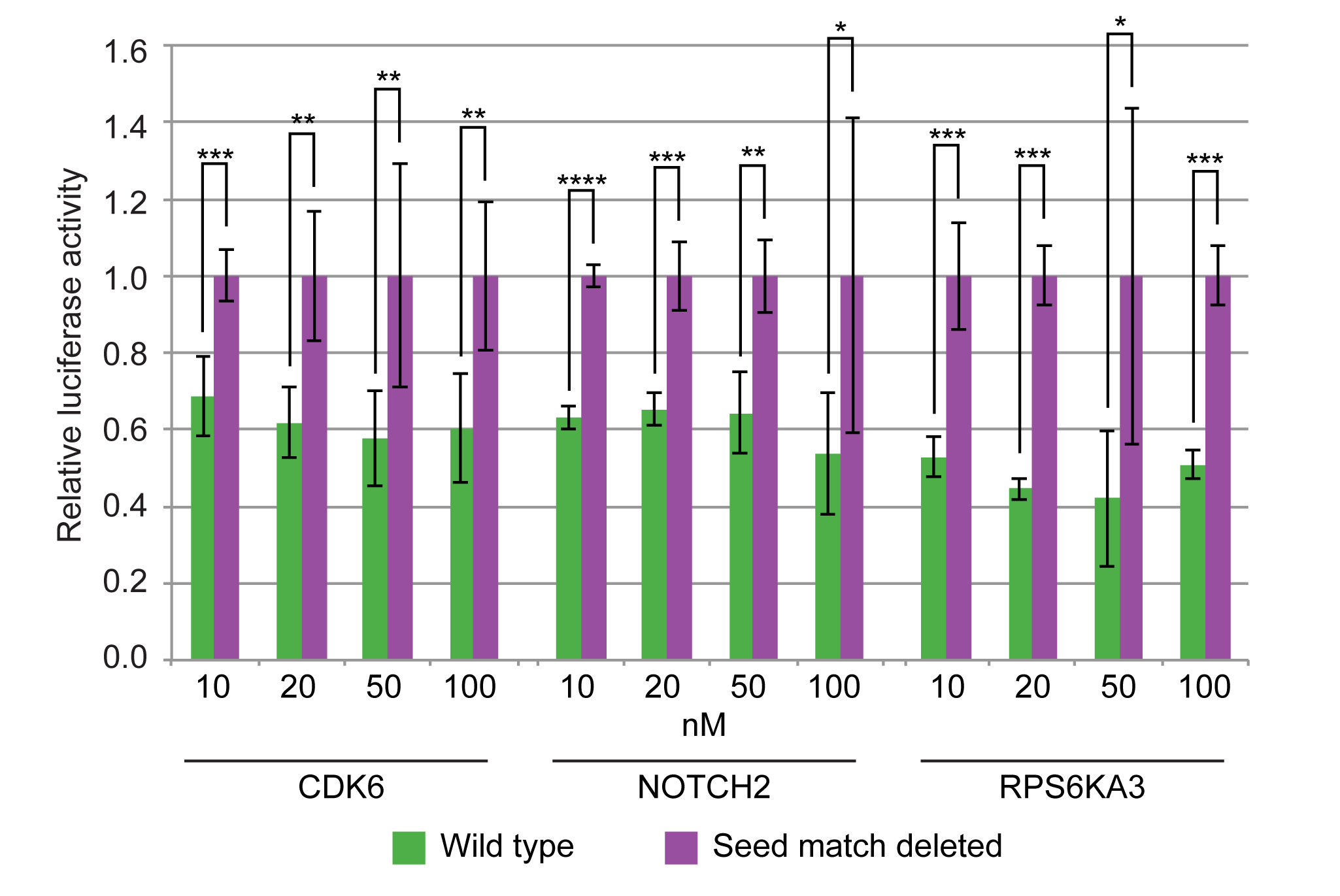

Supplement: S7 Fig — Luciferase reporter assays showed miR-191 directly targets the 3’ UTRs of the genes indicated on the X-axis. Varying concentrations of miR-191 were used, and final concentrations of miR-191 are indicated on the X-axis. The Y-axis denotes relative luciferase units from miR-191 transfected HEK293 cells normalized to Control siRNA transfected cells. Purple bars: 3’ UTRs with the putative miR-191 target site deleted. Green bars: Intact 3’ UTRs. Bars indicate the mean, and error bars denote ± SD, n = 3. P-values were estimated by Student’s one tailed t-test comparing miR-191 normalized to Control siRNA luciferase activity with intact 3’ UTRs to luciferase activity with miR-191 target site deleted 3’ UTRs. *P < 0.05; **P < 0.01; ***P < 0.001; ****P < 0.0001. (TIF) [file pone.0126535.s007.tif]
