# Supplementary material for: MiR-191 Regulates Primary Human Fibroblast Proliferation and Directly Targets Multiple Oncogenes
Source: PLoS One. 2015 May 20;10(5):e0126535. doi: 10.1371/journal.pone.0126535 (PMC4439112; doi:10.1371/journal.pone.0126535)
Supplement: S1 Table — (DOCX) [file pone.0126535.s008.docx]

| GENE | TARGET RANK SCORE | ENRICHMENT | REPRESSION |
| --- | --- | --- | --- |
| HRSP12 | 7.36 | 11.39 | 3.32 |
| TMEM70 | 5.36 | 8.49 | 2.23 |
| MRPS7 | 4.84 | 7.91 | 1.78 |
| MCM5 | 4.79 | 8.07 | 1.5 |
| BCAT2 | 4.52 | 6.48 | 2.55 |
| WDFY1 | 4.36 | 6.38 | 2.34 |
| GPR89B | 4.12 | 6.5 | 1.74 |
| SGPP1 | 4.11 | 5.52 | 2.71 |
| PUS3 | 3.93 | 6.65 | 1.21 |
| GPR89A | 3.9 | 6.13 | 1.67 |
| MPST | 3.83 | 5.92 | 1.74 |
| GPR89C | 3.66 | 5.72 | 1.61 |
| LRRC47 | 3.64 | 5.61 | 1.68 |
| TMED10 | 3.6 | 5.81 | 1.4 |
| LRRC8A | 3.5 | 4.8 | 2.2 |
| RARG | 3.37 | 4.47 | 2.27 |
| ADCK1 | 3.37 | 5.48 | 1.26 |
| RGS2 | 3.28 | 4.56 | 2 |
| CCDC127 | 3.26 | 5.18 | 1.34 |
| EIF1B | 3.25 | 5.54 | 0.97 |
| C18orf32 | 3.25 | 4.61 | 1.88 |
| GORAB | 3.21 | 5.1 | 1.31 |
| FAM82A2 | 3.18 | 4.11 | 2.24 |
| B4GALNT1 | 3.13 | 5.23 | 1.04 |
| SMCR7L | 3.12 | 4.53 | 1.71 |
| CSDA | 3.11 | 4.47 | 1.74 |
| PCSK9 | 3.1 | 4.3 | 1.89 |
| DDT | 3.08 | 4.83 | 1.32 |
| DCAF15 | 3.03 | 4.57 | 1.49 |
| GLUD1 | 2.95 | 4.66 | 1.23 |
| ZNF124 | 2.94 | 4.67 | 1.21 |
| RPL9 | 2.93 | 4.61 | 1.24 |
| TMEM38B | 2.9 | 4.11 | 1.7 |
| DHCR24 | 2.86 | 3.95 | 1.76 |
| VPS72 | 2.83 | 4.32 | 1.34 |
| BNIP1 | 2.82 | 4.12 | 1.53 |
| IPPK | 2.81 | 3.77 | 1.86 |
| RPS6KA1 | 2.79 | 4.03 | 1.56 |
| SLC37A4 | 2.77 | 3.72 | 1.81 |
| SSR1 | 2.75 | 4.43 | 1.06 |
| C9orf85 | 2.74 | 4.41 | 1.06 |
| FN3KRP | 2.72 | 4.09 | 1.35 |
| PHC2 | 2.69 | 3.8 | 1.57 |
| SERF1B | 2.69 | 3.84 | 1.54 |
| BRMS1L | 2.67 | 3.16 | 2.19 |
| ZCCHC8 | 2.67 | 3.98 | 1.37 |
| TSPAN13 | 2.66 | 3.12 | 2.2 |
| PRMT6 | 2.64 | 3.8 | 1.48 |
| SLC38A7 | 2.63 | 3.09 | 2.18 |
| TMEM134 | 2.63 | 4.26 | 1 |
| HEY1 | 2.63 | 3.92 | 1.33 |
| MIF4GD | 2.63 | 3.87 | 1.39 |
| NOP58 | 2.62 | 4.17 | 1.07 |
| CEBPB | 2.59 | 3.98 | 1.2 |
| ZSWIM1 | 2.55 | 3.75 | 1.36 |
| KTI12 | 2.52 | 3.89 | 1.16 |
| SLC25A24 | 2.52 | 2.89 | 2.15 |
| TTC9C | 2.51 | 3.74 | 1.27 |
| FTSJD1 | 2.5 | 3.22 | 1.77 |
| C14orf126 | 2.5 | 3.26 | 1.73 |
| AGPAT2 | 2.5 | 3.37 | 1.62 |
| TOMM5 | 2.48 | 3.71 | 1.24 |
| DUSP7 | 2.48 | 2.65 | 2.3 |
| BCAR1 | 2.48 | 3.96 | 1.01 |
| WWP1 | 2.47 | 2.68 | 2.26 |
| DBP | 2.46 | 3.69 | 1.22 |
| BDNF | 2.41 | 3.65 | 1.17 |
| CDK9 | 2.39 | 3.52 | 1.27 |
| ZDHHC5 | 2.39 | 3.38 | 1.41 |
| GATAD2A | 2.39 | 3.09 | 1.7 |
| BICD2 | 2.38 | 2.99 | 1.77 |
| POC1B | 2.37 | 3.48 | 1.27 |
| MT1F | 2.37 | 2.35 | 2.39 |
| RNPS1 | 2.37 | 3.57 | 1.17 |
| C19orf21 | 2.36 | 3.17 | 1.55 |
| NUDT5 | 2.36 | 3.56 | 1.17 |
| FAM13C | 2.36 | 3.04 | 1.68 |
| URM1 | 2.35 | 3.3 | 1.4 |
| COX6A1 | 2.34 | 3.57 | 1.1 |
| FBXL14 | 2.31 | 3.21 | 1.42 |
| IFFO2 | 2.31 | 2.98 | 1.65 |
| LIN54 | 2.31 | 3.28 | 1.33 |
| RIC8B | 2.31 | 3.43 | 1.19 |
| CDC123 | 2.3 | 3.36 | 1.24 |
| MTMR12 | 2.3 | 3.01 | 1.59 |
| GMFB | 2.29 | 2.57 | 2.02 |
| DCTN4 | 2.29 | 3.17 | 1.41 |
| TMED7 | 2.29 | 3.23 | 1.34 |
| NAA38 | 2.27 | 3.42 | 1.12 |
| HMGN1 | 2.26 | 3.39 | 1.12 |
| ZNF527 | 2.26 | 3.51 | 1 |
| C8orf82 | 2.26 | 2.68 | 1.83 |
| SLC7A1 | 2.25 | 3.05 | 1.45 |
| C11orf68 | 2.24 | 3.06 | 1.41 |
| FZD5 | 2.23 | 2.87 | 1.58 |
| HIST1H3A | 2.23 | 2.99 | 1.47 |
| MICA | 2.23 | 2.62 | 1.85 |
| DUSP3 | 2.23 | 2.74 | 1.72 |
| MAPK8IP1 | 2.23 | 2.68 | 1.78 |
| CASP1 | 2.22 | 3.34 | 1.1 |
| FAM109B | 2.22 | 2.99 | 1.46 |
| AK3 | 2.21 | 2.81 | 1.61 |
| ATRN | 2.21 | 3.25 | 1.18 |
| C11orf61 | 2.2 | 3.18 | 1.22 |
| LIN7B | 2.19 | 2.57 | 1.81 |
| CLN3 | 2.19 | 3.23 | 1.15 |
| ZNF275 | 2.18 | 2.37 | 2 |
| RAB4A | 2.16 | 2.62 | 1.7 |
| NEURL4 | 2.16 | 3.19 | 1.13 |
| NFKB1 | 2.15 | 3.15 | 1.15 |
| NUTF2 | 2.15 | 3.06 | 1.25 |
| ENTPD6 | 2.15 | 2.85 | 1.45 |
| CHMP6 | 2.14 | 3.28 | 1 |
| NMRAL1 | 2.13 | 2.96 | 1.31 |
| FOPNL | 2.13 | 2.49 | 1.76 |
| CCDC90A | 2.12 | 2.91 | 1.34 |
| PRR4 | 2.12 | 2.22 | 2.01 |
| CPE | 2.11 | 2.93 | 1.29 |
| ORC3 | 2.11 | 2.79 | 1.43 |
| HIST2H2BF | 2.1 | 3.05 | 1.15 |
| BIRC3 | 2.1 | 2.67 | 1.52 |
| TICAM2 | 2.09 | 3.18 | 1 |
| FAM45A | 2.09 | 2.73 | 1.46 |
| ZBTB2 | 2.09 | 2.9 | 1.29 |
| PPP1CB | 2.07 | 2.51 | 1.62 |
| VTI1B | 2.07 | 2.88 | 1.26 |
| GLIPR2 | 2.06 | 2.62 | 1.49 |
| SCYL1 | 2.06 | 2.83 | 1.28 |
| LEPROTL1 | 2.06 | 2.54 | 1.57 |
| C9orf89 | 2.04 | 3.07 | 1.02 |
| C1QTNF6 | 2.04 | 2.63 | 1.44 |
| RPS6KA3 | 2.04 | 2.46 | 1.63 |
| TAF5 | 2.03 | 2.4 | 1.67 |
| RCC2 | 2.02 | 2.46 | 1.57 |
| C14orf93 | 2.02 | 2.11 | 1.93 |
| DUS2L | 2.01 | 2.54 | 1.48 |
| VEGFB | 2.01 | 3 | 1.03 |
| ATP2A2 | 2 | 2.97 | 1.04 |
| SAFB | 2 | 2.97 | 1.02 |
| WSB2 | 1.99 | 2.67 | 1.31 |
| ZNF26 | 1.99 | 2.62 | 1.36 |
| P2RY2 | 1.99 | 2.77 | 1.21 |
| STARD7 | 1.97 | 2.62 | 1.33 |
| C17orf51 | 1.96 | 2.53 | 1.38 |
| TFPI2 | 1.96 | 2.93 | 0.98 |
| GSTT2 | 1.96 | 1.73 | 2.19 |
| OXSR1 | 1.95 | 2.31 | 1.59 |
| TCF7L2 | 1.95 | 2.8 | 1.09 |
| KLF7 | 1.95 | 2.2 | 1.69 |
| MPP7 | 1.95 | 2.48 | 1.42 |
| FRMD8 | 1.94 | 2.21 | 1.67 |
| HOXA13 | 1.94 | 2.95 | 0.94 |
| BLOC1S1 | 1.94 | 2.52 | 1.36 |
| DPY19L4 | 1.94 | 2.14 | 1.74 |
| RTN4R | 1.93 | 1.88 | 1.98 |
| ULK4 | 1.93 | 2.52 | 1.34 |
| SBDS | 1.92 | 2.39 | 1.46 |
| NGFRAP1 | 1.92 | 2.61 | 1.23 |
| PSMG3 | 1.92 | 2.86 | 0.97 |
| LY6G5B | 1.92 | 1.79 | 2.04 |
| KLHDC10 | 1.92 | 2.31 | 1.53 |
| TMEM39B | 1.92 | 2.91 | 0.94 |
| API5 | 1.91 | 1.95 | 1.87 |
| MESDC2 | 1.91 | 2.54 | 1.28 |
| TOB2 | 1.9 | 2.23 | 1.58 |
| TLE1 | 1.9 | 2.4 | 1.39 |
| TSC22D3 | 1.9 | 2.68 | 1.12 |
| TRMT6 | 1.9 | 2.09 | 1.71 |
| OCRL | 1.89 | 2.59 | 1.2 |
| FAM89A | 1.89 | 1.97 | 1.82 |
| PPT2 | 1.88 | 1.95 | 1.81 |
| CALCOCO1 | 1.88 | 2.28 | 1.47 |
| PARP9 | 1.88 | 2.46 | 1.31 |
| RMND5A | 1.88 | 1.71 | 2.06 |
| C7orf26 | 1.88 | 2.61 | 1.15 |
| ENTPD5 | 1.88 | 2.21 | 1.55 |
| INTS5 | 1.88 | 2.57 | 1.2 |
| AGBL5 | 1.87 | 2.63 | 1.12 |
| ARL17A | 1.87 | 1.93 | 1.81 |
| PRR14 | 1.87 | 2.29 | 1.45 |
| CREB3L4 | 1.87 | 2.75 | 0.99 |
| CKLF | 1.86 | 2.44 | 1.27 |
| ANKLE2 | 1.86 | 2.55 | 1.16 |
| SDC1 | 1.86 | 2.21 | 1.52 |
| FAM136A | 1.86 | 2.26 | 1.46 |
| PLEK2 | 1.85 | 2.63 | 1.08 |
| YPEL5 | 1.85 | 2.43 | 1.28 |
| TGIF2 | 1.85 | 2.37 | 1.32 |
| RNF8 | 1.84 | 2.25 | 1.43 |
| CDCA7L | 1.84 | 2.34 | 1.34 |
| TBC1D2B | 1.84 | 2.28 | 1.4 |
| YIPF5 | 1.84 | 2.25 | 1.44 |
| HLTF | 1.83 | 2.19 | 1.46 |
| ABHD3 | 1.83 | 2.49 | 1.18 |
| ZIC2 | 1.83 | 2.36 | 1.3 |
| CTDSP2 | 1.83 | 2.27 | 1.39 |
| C15orf17 | 1.83 | 2.32 | 1.35 |
| TBPL1 | 1.82 | 2.26 | 1.37 |
| MFAP3 | 1.82 | 2.11 | 1.52 |
| PI4KB | 1.82 | 2.29 | 1.35 |
| RDH10 | 1.82 | 2.22 | 1.43 |
| PARS2 | 1.81 | 2.27 | 1.35 |
| FAM131A | 1.81 | 2.3 | 1.33 |
| RNF212 | 1.81 | 2.34 | 1.28 |
| SPAG9 | 1.81 | 2.25 | 1.37 |
| FAM195B | 1.8 | 1.8 | 1.8 |
| SLC39A13 | 1.8 | 1.57 | 2.02 |
| RPN1 | 1.8 | 2.26 | 1.34 |
| MAPRE3 | 1.8 | 2.54 | 1.05 |
| DCTN3 | 1.8 | 2.39 | 1.2 |
| METAP1D | 1.79 | 2.49 | 1.08 |
| FYN | 1.79 | 2.28 | 1.29 |
| ADO | 1.79 | 2.26 | 1.33 |
| MAP2K3 | 1.79 | 2.4 | 1.18 |
| GTDC1 | 1.78 | 2.06 | 1.51 |
| GAGE12I | 1.78 | 1.78 | 1.78 |
| ANKRD52 | 1.78 | 2 | 1.57 |
| PDGFC | 1.78 | 2.15 | 1.42 |
| COPS7A | 1.78 | 2.34 | 1.22 |
| ARFIP1 | 1.77 | 1.81 | 1.72 |
| CSTF1 | 1.77 | 2.33 | 1.21 |
| RWDD2A | 1.76 | 1.74 | 1.77 |
| LIMK1 | 1.76 | 1.9 | 1.63 |
| SCFD2 | 1.76 | 2.42 | 1.09 |
| SOX4 | 1.76 | 2.31 | 1.22 |
| LDHA | 1.76 | 2.48 | 1.04 |
| ANKRD27 | 1.75 | 2 | 1.49 |
| LRRC27 | 1.74 | 2.26 | 1.21 |
| RTN4IP1 | 1.74 | 2.5 | 0.98 |
| DDX20 | 1.74 | 2.46 | 1.01 |
| HOXD8 | 1.74 | 2.18 | 1.3 |
| WBP1 | 1.74 | 1.96 | 1.52 |
| SPOP | 1.74 | 2.26 | 1.23 |
| C16orf52 | 1.73 | 1.98 | 1.47 |
| RNF19B | 1.73 | 2.38 | 1.09 |
| EARS2 | 1.73 | 2.08 | 1.38 |
| C12orf49 | 1.73 | 2.07 | 1.39 |
| SNX2 | 1.73 | 2.25 | 1.22 |
| TLX3 | 1.73 | 2.46 | 1 |
| PTBP1 | 1.73 | 2.4 | 1.07 |
| MED25 | 1.73 | 2.06 | 1.39 |
| ZNF772 | 1.73 | 2.09 | 1.38 |
| PPAPDC2 | 1.73 | 2.27 | 1.18 |
| SMYD3 | 1.72 | 2.05 | 1.39 |
| MOV10 | 1.72 | 2.09 | 1.35 |
| ASF1A | 1.71 | 1.97 | 1.45 |
| GNL3L | 1.71 | 1.81 | 1.61 |
| LAGE3 | 1.71 | 2.15 | 1.27 |
| NPLOC4 | 1.71 | 2.17 | 1.25 |
| GLTPD1 | 1.71 | 2.28 | 1.13 |
| ZNF330 | 1.71 | 2.27 | 1.14 |
| ATG9A | 1.71 | 2.31 | 1.1 |
| ANG | 1.71 | 1.07 | 2.34 |
| ZFAND2A | 1.71 | 2.29 | 1.13 |
| FZD3 | 1.7 | 2.27 | 1.13 |
| AGPHD1 | 1.7 | 2.04 | 1.36 |
| CSF1 | 1.7 | 2.14 | 1.26 |
| C18orf56 | 1.7 | 2.57 | 0.83 |
| TRAPPC10 | 1.7 | 2.05 | 1.35 |
| OSBPL8 | 1.69 | 2.14 | 1.24 |
| TRAK2 | 1.69 | 2.29 | 1.09 |
| TBC1D14 | 1.69 | 2.16 | 1.22 |
| DYRK2 | 1.69 | 1.96 | 1.42 |
| YME1L1 | 1.69 | 2.19 | 1.18 |
| GCNT2 | 1.68 | 1.73 | 1.64 |
| NT5C | 1.68 | 2.03 | 1.33 |
| RRP7A | 1.68 | 2.06 | 1.31 |
| KANK1 | 1.68 | 2.06 | 1.31 |
| C4orf46 | 1.68 | 1.8 | 1.55 |
| TMEM30A | 1.68 | 1.96 | 1.4 |
| GAGE2C | 1.68 | 1.93 | 1.42 |
| PHF13 | 1.68 | 1.95 | 1.41 |
| STK19 | 1.68 | 0.93 | 2.42 |
| ARHGAP12 | 1.68 | 1.8 | 1.56 |
| DONSON | 1.68 | 2.09 | 1.27 |
| CEP164 | 1.68 | 1.99 | 1.38 |
| INPP1 | 1.67 | 2.42 | 0.92 |
| VRK3 | 1.67 | 1.99 | 1.34 |
| PLEKHO2 | 1.67 | 2.05 | 1.28 |
| CP | 1.67 | 2.34 | 1 |
| SCOC | 1.67 | 1.85 | 1.5 |
| IL17RC | 1.67 | 2.03 | 1.32 |
| LOC100505679 | 1.67 | 1.67 | 1.67 |
| ATG16L1 | 1.66 | 2.08 | 1.25 |
| TSEN2 | 1.66 | 2.1 | 1.22 |
| RAB4B | 1.66 | 1.53 | 1.79 |
| NOTCH2 | 1.66 | 1.78 | 1.53 |
| ADIPOR1 | 1.66 | 1.97 | 1.36 |
| IDI2 | 1.66 | 1.8 | 1.52 |
| PDE7A | 1.66 | 1.85 | 1.47 |
| FAM73A | 1.66 | 1.78 | 1.53 |
| GAGE2E | 1.66 | 1.88 | 1.45 |
| MED20 | 1.66 | 2.11 | 1.2 |
| DDHD1 | 1.65 | 1.83 | 1.47 |
| ZNF174 | 1.65 | 2 | 1.31 |
| GSG2 | 1.65 | 2.2 | 1.1 |
| ZXDB | 1.65 | 1.9 | 1.39 |
| CAPZA1 | 1.65 | 1.87 | 1.42 |
| PNPO | 1.65 | 2.02 | 1.27 |
| IFITM1 | 1.65 | 1.89 | 1.41 |
| ALDH18A1 | 1.64 | 2.04 | 1.24 |
| ZNF419 | 1.64 | 2.19 | 1.1 |
| TRAM2 | 1.64 | 1.97 | 1.31 |
| B3GALTL | 1.64 | 2.07 | 1.21 |
| PAFAH1B1 | 1.64 | 1.98 | 1.3 |
| IST1 | 1.64 | 2 | 1.28 |
| SAMD1 | 1.64 | 1.95 | 1.32 |
| BCDIN3D | 1.64 | 2.17 | 1.1 |
| MAPK9 | 1.64 | 1.91 | 1.37 |
| EPHX1 | 1.64 | 2.27 | 1 |
| EFNB2 | 1.63 | 1.91 | 1.35 |
| GRSF1 | 1.63 | 2.1 | 1.15 |
| PRKAG2 | 1.63 | 2.09 | 1.16 |
| LAMTOR1 | 1.62 | 1.89 | 1.34 |
| AASDHPPT | 1.62 | 1.85 | 1.39 |
| TACC2 | 1.62 | 1.98 | 1.26 |
| CADM1 | 1.62 | 1.73 | 1.51 |
| C9orf5 | 1.62 | 1.55 | 1.69 |
| SLC6A8 | 1.62 | 1.8 | 1.44 |
| NUP133 | 1.62 | 2.1 | 1.13 |
| PGM2L1 | 1.62 | 1.9 | 1.35 |
| ISY1 | 1.62 | 1.87 | 1.38 |
| C5orf15 | 1.62 | 1.77 | 1.46 |
| MPDU1 | 1.61 | 1.88 | 1.34 |
| TPD52L2 | 1.61 | 1.91 | 1.3 |
| CDK17 | 1.61 | 2.08 | 1.14 |
| ZNF274 | 1.61 | 2.06 | 1.16 |
| HEATR6 | 1.61 | 2 | 1.23 |
| HEATR1 | 1.61 | 2.1 | 1.13 |
| WIZ | 1.61 | 2.1 | 1.13 |
| VANGL1 | 1.61 | 1.97 | 1.26 |
| PIGA | 1.6 | 1.65 | 1.56 |
| TFAP4 | 1.6 | 2.1 | 1.11 |
| TEX261 | 1.6 | 2.12 | 1.08 |
| ALOX5AP | 1.6 | 1.85 | 1.36 |
| PABPN1 | 1.6 | 1.92 | 1.28 |
| MAPK6 | 1.6 | 1.57 | 1.64 |
| C2orf29 | 1.6 | 1.74 | 1.45 |
| PRDM11 | 1.6 | 2.23 | 0.96 |
| SAP130 | 1.59 | 1.97 | 1.21 |
| EIF1 | 1.59 | 2.28 | 0.9 |
| CCNE1 | 1.59 | 1.5 | 1.67 |
| C9orf16 | 1.59 | 1.51 | 1.66 |
| ABCB6 | 1.59 | 2.36 | 0.83 |
| STAT5B | 1.59 | 1.82 | 1.36 |
| MAX | 1.59 | 1.89 | 1.3 |
| WDR85 | 1.59 | 2.16 | 1.02 |
| LMBR1L | 1.58 | 1.42 | 1.74 |
| SPATA24 | 1.58 | 1.98 | 1.19 |
| ATP5H | 1.58 | 2.11 | 1.05 |
| PI4KA | 1.58 | 2.18 | 0.98 |
| WDR45L | 1.58 | 1.9 | 1.25 |
| MT2A | 1.58 | 1.86 | 1.29 |
| ZNF22 | 1.58 | 1.81 | 1.36 |
| HS6ST1 | 1.58 | 2.15 | 1.01 |
| STK35 | 1.58 | 1.78 | 1.38 |
| CIB1 | 1.58 | 1.77 | 1.39 |
| NDST2 | 1.58 | 1.94 | 1.22 |
| MICB | 1.58 | 1.56 | 1.61 |
| ZNRF2 | 1.58 | 1.99 | 1.17 |
| RXRA | 1.58 | 1.94 | 1.23 |
| NFATC2IP | 1.57 | 1.71 | 1.44 |
| C5orf43 | 1.57 | 2.13 | 1.01 |
| RSU1 | 1.57 | 1.92 | 1.22 |
| SNX6 | 1.57 | 1.72 | 1.42 |
| HOMEZ | 1.57 | 2.11 | 1.03 |
| C20orf29 | 1.57 | 1.88 | 1.26 |
| TM9SF4 | 1.57 | 1.98 | 1.17 |
| MOSPD3 | 1.57 | 1.46 | 1.67 |
| ENC1 | 1.57 | 2.14 | 0.99 |
| EXOC4 | 1.57 | 2 | 1.13 |
| BEND3 | 1.57 | 2.16 | 0.99 |
| CLEC16A | 1.57 | 2.16 | 0.98 |
| ETHE1 | 1.56 | 2.01 | 1.11 |
| FUBP3 | 1.56 | 2.07 | 1.05 |
| SCHIP1 | 1.56 | 1.85 | 1.26 |
| MOB3A | 1.56 | 1.84 | 1.28 |
| ZBTB10 | 1.56 | 1.62 | 1.49 |
| MORF4L2 | 1.56 | 1.49 | 1.62 |
| LBH | 1.56 | 2.12 | 1 |
| CCNI | 1.56 | 1.42 | 1.7 |
| ZBTB48 | 1.56 | 1.92 | 1.2 |
| NASP | 1.56 | 1.99 | 1.12 |
| UBE3B | 1.56 | 1.92 | 1.19 |
| PSMF1 | 1.56 | 2.17 | 0.96 |
| PLAU | 1.56 | 2.13 | 1 |
| CETN2 | 1.55 | 1.66 | 1.44 |
| RDM1 | 1.55 | 1.55 | 1.55 |
| ACAD10 | 1.55 | 2.09 | 1 |
| PTPN21 | 1.55 | 2.1 | 1 |
| PRRG1 | 1.55 | 1.75 | 1.35 |
| VEZF1 | 1.55 | 1.86 | 1.24 |
| SLC15A4 | 1.55 | 1.93 | 1.17 |
| PIAS2 | 1.55 | 1.85 | 1.24 |
| TET3 | 1.55 | 1.64 | 1.45 |
| CLIP1 | 1.55 | 1.63 | 1.47 |
| LOC285033 | 1.55 | 2.11 | 1 |
| GHDC | 1.54 | 1.76 | 1.32 |
| TNFAIP2 | 1.54 | 1.91 | 1.17 |
| SURF4 | 1.54 | 1.69 | 1.39 |
| PIK3R3 | 1.54 | 1.73 | 1.36 |
| PEF1 | 1.54 | 1.83 | 1.25 |
| SLC25A46 | 1.54 | 1.78 | 1.3 |
| SNX15 | 1.54 | 1.44 | 1.63 |
| C22orf40 | 1.54 | 2.16 | 0.91 |
| TRMT12 | 1.54 | 1.95 | 1.13 |
| TBC1D22B | 1.54 | 1.9 | 1.17 |
| C20orf94 | 1.54 | 1.74 | 1.34 |
| LTBP1 | 1.53 | 1.86 | 1.21 |
| MGST3 | 1.53 | 1.85 | 1.22 |
| ASPHD1 | 1.53 | 2.16 | 0.89 |
| ATP6V0A4 | 1.53 | 2.03 | 1.02 |
| PRPF39 | 1.53 | 1.96 | 1.1 |
| FNIP2 | 1.53 | 1.75 | 1.31 |
| IPMK | 1.53 | 1.72 | 1.34 |
| NBR1 | 1.53 | 1.74 | 1.33 |
| FCHO2 | 1.53 | 1.65 | 1.41 |
| HES7 | 1.53 | 1.43 | 1.64 |
| MAGI3 | 1.53 | 1.76 | 1.3 |
| SPAG4 | 1.53 | 1.53 | 1.53 |
| FXC1 | 1.52 | 1.67 | 1.37 |
| ALCAM | 1.52 | 1.57 | 1.46 |
| ZNF557 | 1.52 | 1.83 | 1.21 |
| SEC11A | 1.52 | 1.88 | 1.17 |
| NXT2 | 1.52 | 1.44 | 1.6 |
| VAMP8 | 1.52 | 1.65 | 1.38 |
| CEP19 | 1.52 | 1.81 | 1.22 |
| LZTFL1 | 1.52 | 1.23 | 1.8 |
| PSME3 | 1.52 | 1.96 | 1.09 |
| B3GNT1 | 1.52 | 1.7 | 1.35 |
| P4HB | 1.52 | 1.91 | 1.13 |
| TJP1 | 1.52 | 1.74 | 1.29 |
| OR51B5 | 1.52 | 1.71 | 1.33 |
| RARS | 1.52 | 1.64 | 1.39 |
| RAB22A | 1.52 | 1.67 | 1.36 |
| AUTS2 | 1.52 | 1.98 | 1.05 |
| KLHL7 | 1.52 | 1.71 | 1.33 |
| GM2A | 1.52 | 1.72 | 1.32 |
| PTAR1 | 1.52 | 1.54 | 1.51 |
| GCNT1 | 1.51 | 1.76 | 1.27 |
| COQ6 | 1.51 | 1.82 | 1.2 |
| TMEM93 | 1.51 | 1.84 | 1.17 |
| IFIT1 | 1.51 | 1.38 | 1.65 |
| CRK | 1.51 | 1.57 | 1.45 |
| DHX40 | 1.51 | 1.86 | 1.16 |
| MPPED2 | 1.51 | 1.65 | 1.38 |
| ZNF532 | 1.51 | 1.88 | 1.14 |
| SLC12A7 | 1.51 | 2.02 | 1 |
| ABCB9 | 1.51 | 1.61 | 1.4 |
| APAF1 | 1.51 | 1.56 | 1.45 |
| TRAF3 | 1.51 | 1.92 | 1.1 |
| SLC10A7 | 1.51 | 1.63 | 1.4 |
| BOK | 1.5 | 1.87 | 1.13 |
| PAPD4 | 1.5 | 1.78 | 1.23 |
| ZNF473 | 1.5 | 1.62 | 1.37 |
| CA2 | 1.5 | 1.54 | 1.47 |
| GLTP | 1.5 | 1.62 | 1.38 |
| INPP5A | 1.5 | 1.66 | 1.33 |
| C15orf58 | 1.5 | 1.81 | 1.19 |
| HMGCR | 1.5 | 1.71 | 1.29 |
| HIST1H2AK | 1.5 | 1.59 | 1.41 |
| BSDC1 | 1.5 | 1.65 | 1.35 |
