# Supplementary material for: MiR-191 Regulates Primary Human Fibroblast Proliferation and Directly Targets Multiple Oncogenes
Source: PLoS One. 2015 May 20;10(5):e0126535. doi: 10.1371/journal.pone.0126535 (PMC4439112; doi:10.1371/journal.pone.0126535)
Supplement: S2 Table — (DOCX) [file pone.0126535.s009.docx]

| Assay | Gene / locus | Primers |
| --- | --- | --- |
|  | AGO2-F | CACCATGTACTCGGGAGCC |
|  | AGO2-R | TTGATTGTTCTCCCGGAGGT |
|  | BCL2-F | CTGAGTACCTGAACCGGCA |
|  | BCL2-R | GAGAAATCAAACAGAGGCCG |
|  | CCNE1-F | TCTTTGTCAGGTGTGGGGA |
|  | CCNE1-R | GAAATGGCCAAAATCGACAG |
|  | CDK6-F | ATGCCGCTCTCCACCAT |
|  | CDK6-R | TGTCTGTTCGTGACACTGTGC |
|  | CDK9-F | AAGGTGCTGATGGAAAACGA |
|  | CDK9-R | CAAGTTGACCACATTCTCGTG |
|  | CHMP6-F | CCATGGGTAACCTGTTCGG |
| qRT-PCR | CHMP6-R | GGTACTGCCTCAGCTTGTCC |
|  | NOTCH2D-F | TGGAGATGACTGCAGTGAGAAC |
|  | NOTCH2D-R | TCATCCAGATGACACAGGAGAC |
|  | PRMT6-F (no introns) | ACGAGTGCTACTCGGACGTT |
|  | PRMT6-R (no introns) | AGTTCCGAAGGATACCCAGG |
|  | RPS6KA3-F | GTGGCAGAAGATGGCTGTG |
|  | RPS6KA3-R | TGGGTTAATCTCCTCCTCTCC |
|  | SLC7A1-F | GTCTGTCTGTTCGCGATCCT |
|  | SLC7A1-R | AGAGGACAGCCTCGATCTTG |
|  | ZBTB2-F | GTTGCAATCGGCGATGTAT |
|  | ZBTB2-R | AGTTGGTTTCAAGCGGACAC |
|  | B2M-F | AGATGAGTATGCCTGCCGTGTGAA |
|  | B2M-R | TGCTGCTTACATGTCTCGATCCCA |
|  | GAPDH-F | CTGGGCTACACTGAGCACCAG |
|  | GAPDH-R | CCAGCGTCAAAGGTGGAG |
|  | AGO2_F_XhoI | GTTTTActcgagAGCGATTGTGTACCGAGTGG |
|  | AGO2_R_NotI | TTCTCTgcggccgcAGACATAACAGTGAAAAAGGATTGT |
|  | BCL2_F_XhoI | TTATCActcgagGATGTAGCTCTGGCCCAGTG |
|  | BCL2_R_NotI | TATAATgcggccgcAATGAGCTATCTGGAGGGCC |
|  | CDK6_F_XhoI | AAAATGctcgagTGTTTCTGCATTGCCATAGGG |
|  | CDK6_R_NotI | CTATCTgcggccgcAAACCCTGGGTCACAAAGCA |
|  | CDK9_F_XhoI | CTATGTctcgagCATCGTGGAGACAGGGCATT |
| Cloning | CDK9_R_NotI | TCTGGTgcggccgcTTGCCAACCTTCCTTCCTGG |
|  | NOTCH2_F_XhoI_DWN | TCATTCctcgagGTGACTCTCTGCCCTTGGAC |
|  | NOTCH2_R_NotI_DWN | ACTTAAgcggccgcACAAACCAATCATTTACATAACAGCA |
|  | PRMT6_F_XhoI | AAAGGGctcgagCACGTGCAAGTAGGGGGAAT |
|  | PRMT6_R_NotI | GCAAAAgcggccgcACTCACAGCACACAAGACTT |
|  | RPS6KA3_F_XhoI | GGTAGCctcgagCTGAGAGACACCTGCAAGCA |
|  | RPS6KA3_R_NotI | ATACTCgcggccgcTCACTTAGCCTGCCCTACCT |
|  | SLC7A1_F_XhoI | CTTGTActcgagAGCCAGAGGAAACCTGAACG |
|  | SLC7A1_R_NotI | AGGCAGgcggccgcGGAGATCTGCTGGGTTTGCT |
|  | AGO2-mut1 | acgtcattgattttattagattcttccagaatgccttccgtc |
|  | AGO2-mut2 | gacggaaggcattctggaagaatctaataaaatcaatgacgt |
|  | BCL2-mut1 | accattgcactgccagctgcactttgagcc |
|  | BCL2-mut2 | ggctcaaagtgcagctggcagtgcaatggt |
|  | CDK6-mut1 | tgcatgttccttaaaggtgcataattattcttatgcttatattgctacag |
|  | CDK6-mut2 | ctgtagcaatataagcataagaataattatgcacctttaaggaacatgca |
|  | CDK9-mut1 | aaaaaaaattcaacttttaattctccaaactgtgcccaggaatgtct |
| Mutagenesis | CDK9-mut2 | agacattcctgggcacagtttggagaattaaaagttgaatttttttt |
|  | NOTCH2-D-mut1 | catcttaactacctttagaatgaaagtaaaaacaaagtgtgcattttcct |
|  | NOTCH2-D-mut2 | aggaaaatgcacactttgtttttactttcattctaaaggtagttaagatg |
|  | PRMT6-mut1 | caattacaaagtccattttacttttaatgtgttcatcaactttagcacc |
|  | PRMT6-mut2 | ggtgctaaagttgatgaacacattaaaagtaaaatggactttgtaattg |
|  | RPS6KA3-mut1 | acaagggatacagtgctaatatattgtttaagctaagtgcttaaca |
|  | RPS6KA3-mut2 | tgttaagcacttagcttaaacaatatattagcactgtatcccttgt |
|  | SLC7A1-mut1 | attctctttgcctatgtagaaaataaaaaaatcaagcaaataactaccagg |
|  | SLC7A1-mut2 | cctggtagttatttgcttgatttttttattttctacataggcaaagagaat |
