# Supplementary material for: MiR-191 Regulates Primary Human Fibroblast Proliferation and Directly Targets Multiple Oncogenes
Source: PLoS One. 2015 May 20;10(5):e0126535. doi: 10.1371/journal.pone.0126535 (PMC4439112; doi:10.1371/journal.pone.0126535)
Supplement: S3 Table — Spearman rank correlation between RNA-seq FPKM and microarray intensity value for each gene profiled by both RNA-seq and FPKM. For all conditions, the FPKM and microarray intensity values of the biological replicates were averaged for each gene profiled. Values found in the table are the Spearman rank correlation coefficients calculated comparing the averaged RNA-seq FPKM and microarray intensity values. (DOCX) [file pone.0126535.s010.docx]

|  | miR-191 | Mock transfection | Control siRNA |
| --- | --- | --- | --- |
| RIP | 0.71 | 0.72 | 0.73 |
| Total RNA | 0.73 | 0.72 | NA |
